# Characterization and structure‐guided engineering of the novel versatile terpene monooxygenase CYP109Q5 from *Chondromyces apiculatus* DSM436

**DOI:** 10.1111/1751-7915.13354

**Published:** 2018-12-27

**Authors:** Jan M. Klenk, Paulina Dubiel, Mahima Sharma, Gideon Grogan, Bernhard Hauer

**Affiliations:** ^1^ Institute of Biochemistry and Technical Biochemistry Department of Technical Biochemistry University of Stuttgart Allmandring 31 70569 Stuttgart Germany; ^2^ Department of Chemistry University of York Heslington York YO10 5DD UK

## Abstract

One of the major challenges in chemical synthesis is the selective oxyfunctionalization of non‐activated C‐H bonds, which can be enabled by biocatalysis using cytochrome P450 monooxygenases. In this study, we report on the characterization of the versatile CYP109Q5 from *Chondromyces apiculatus* DSM436, which is able to functionalize a wide range of substrates (terpenes, steroids and drugs), including the ring of β‐ionone in non‐allylic positions. The crystal structure of CYP109Q5 revealed flexibility within the active site pocket that permitted the accommodation of bulky substrates, and enabled a structure‐guided approach to engineering the enzyme. Some variants of CYP109Q5 displayed a switch in selectivity towards the non‐allylic positions of β‐ionone, allowing the simultaneous production of 2‐ and 3‐hydroxy‐β‐ionone, which are chemically challenging to synthesize and are important precursors for carotenoid synthesis. An efficient whole‐cell system finally enabled the production of up to 0.5 g l^−1^ hydroxylated products of β‐ionone; this system can be applied to product identification in further biotransformations. Overall, CYP109Q5 proved to be highly evolvable and active. The studies in this work demonstrate that, using rational mutagenesis, the highly versatile CYP109Q5 generalist can be progressively evolved to be an industrially valuable specialist for the synthesis of specific products.

## Introduction

In modern oxidative biocatalysis, the enzyme class of cytochrome P450 monooxygenases (P450s) represents an environmentally friendly alternative to chemical synthesis, which often proceeds under harsh reaction conditions and with limited selectivity (Urlacher and Girhard, 2012). With more than 5000 cloned members, P450s represent a large enzyme superfamily that can be found in all kingdoms of life (Nelson, 2011). The ability to integrate molecular oxygen selectively into non‐activated carbon–hydrogen bonds under mild reaction conditions (RT, atmospheric pressure) makes P450 monooxygenases especially attractive for applications in chemical syntheses (Breuer *et al*., 2004; Urlacher and Girhard, 2012). In nature, P450s play a crucial role in cell metabolism, for example, in terpenoid biosynthesis. Here, terpene molecules are functionalized by P450s, thereby creating a greater diversity in the organisms (Janocha *et al*., 2015). Through the variability of evolved P450 enzymes (over 50 000 sequences), nature provides strategies to oxyfunctionalize specific terpene molecules that can be further improved for implementation into natural or synthetic pathways. However, plant or fungal P450s are often difficult to express, and often possess low activities, thus an increasing number of microbial enzymes are necessary to expand the P450 toolbox in terpene chemistry (Urlacher and Schmid, 2006).

In recent times, numerous concepts have been developed in order to identify new biocatalysts with desirable properties (Bornscheuer *et al*., 2012; Davids *et al*., 2013). The classical strategy is to search for organisms that can already catalyse the desired reaction, either to identify the responsible enzymes for heterologous expression or to adapt the organism to better performance by strain engineering. Furthermore, the number of available P450 sequences that are involved in diverse metabolic pathways is increasing due to the rapid sequencing of genomes (Urlacher and Girhard, 2012). Metagenomic libraries can also be exploited to rapidly display a wide variety of different naturally occurring enzymes to screen for desired properties (Cook *et al*., 2016). The constant progress in gene synthesis technology accelerates this strategy, since codon‐optimized variants of the genes are thus readily available for many expression systems. Particularly promising, however, is a combination of this concept with the classical search among organisms in order to identify wild‐type enzymes. Organisms with special secondary metabolism, such as the myxobacterium *Sorangium cellulosum* So ce56, can serve as interesting sources for P450 monooxygenases (Shimkets *et al*., 2006; Schneiker *et al*., 2007; Khatri *et al*., 2011b). Based on already characterized P450s, the search for homologous enzymes in databases may also lead to a more targeted metagenome analysis. Closely related P450s with an amino acid sequence identity > 40% can be grouped into families and often possess similar functions due to their high conservation in the active site (Scheps *et al*., 2011). In addition to new biocatalysts, protein engineering provides opportunities to extend the application spectrum of previously known P450s to modify enzyme properties such as selectivity, activity or substrate specificity (Whitehouse *et al*., 2012; Hoffmann *et al*., 2016).

Terpenes represent a large, versatile class of natural products whose structural diversity undergoes further diversification by the incorporation of functionalities such as oxyfunctionalization. In recent years, research attempted to find bacterial P450 enzymes for the production of such terpenoids for the flavour and fragrance industry (Daviet and Schalk, 2010). However, despite this, the functionalization of terpenes like sesquiterpenes (premnaspirodiene or bisabolene), or the non‐allylic hydroxylation of the ionone ring of norisoprenoids like β‐ionone, which would lead to high‐value products such as 3‐hydroxy‐β‐ionone, β‐bisabolol or the antifungal phytoalexin solavetivone, is rarely or not yet realized. The extension of the putative biocatalyst spectrum to access valuable or even novel terpenoids is thus highly desired in the flavour and fragrance industry.

In this study, we aimed to enlarge the set of P450 biocatalysts for terpene oxyfunctionalization. Here, we report on the identification, expression, biochemical and structural characterization as well as enzyme engineering of CYP109Q5 from the myxobacterium *Chondromyces apiculatus* DSM436. This enzyme was able to functionalize a diverse set of substrates ranging from monoterpenes over sesquiterpenes to steroids. By applying enzyme engineering based on the crystal structure of CYP109Q5, regioselectivity was shifted towards to the rarely observed non‐allylic oxyfunctionalization of β‐ionone. Finally, an efficient whole‐cell system was developed, to give access to the hydroxylated β‐ionone products.

## Results

### Identification, expression and purification of CYP109Q5

Myxobacteria such as *S. cellulosum* have already been described as a source of a vast variety of different P450s, some of which exhibit activities towards terpenes. Therefore, we searched for myxobacteria related to *S. cellulosum* for which the genome sequences had been determined. Hereby, we chose *Chondromyces apiculatus* DSM436, which harbours 25 putative and yet uncharacterized P450s, suggesting excellent potential for enzyme discovery (Sharma *et al*., 2018). In particular, one P450, attributed to the CYP109 family, was of interest because related P450s have been shown to hydroxylate terpenes (Table S1). *C. apiculatus* DSM436 was obtained from the DSMZ (DSM14612) and the genomic DNA isolated from cells grown on a VY/2 agar plate. The gene of CYP109Q5 was amplified by corresponding primers using PCR, purified on agarose gel and cloned using restriction sites into pET‐28a(+). A more abundant stop codon for *E. coli* was also integrated with the aid of the amplification primers. However, only minor amounts of functional P450 could be expressed using *E. coli* BL21(DE3) or Rosetta(DE3). Accordingly, the genes were cloned into the alternative expression vectors pCWori(+) and pBAD33 to examine the expression. Finally, despite the high GC content and the numerous rare codons in the gene, high expression of 1.33 μM (cell culture) functional P450 could be achieved for CYP109Q5 using the pBAD33 vector in *E. coli* JW5510. For the following characterization, negatively charged CYP109Q5 was partially purified *via* anion exchange chromatography with a sodium chloride step gradient since no purification tag was present and subsequently analysed by SDS‐PAGE (Figs. S1 and S2). CO differential spectroscopy of the reduced protein revealed a typical Soret band at 450 nm of the sodium dithionate reduced P450 and a slight maximum at 420 nm belonging to non‐correctly folded CYP109Q5 (Fig. S2). The usage of the arabinose‐inducible pBAD system has thus enabled sufficient expression of functional CYP109Q5 which opened the possibility of further investigations.

### Reconstitution of P450 activity and determination of substrate spectrum

**Figure 1:**
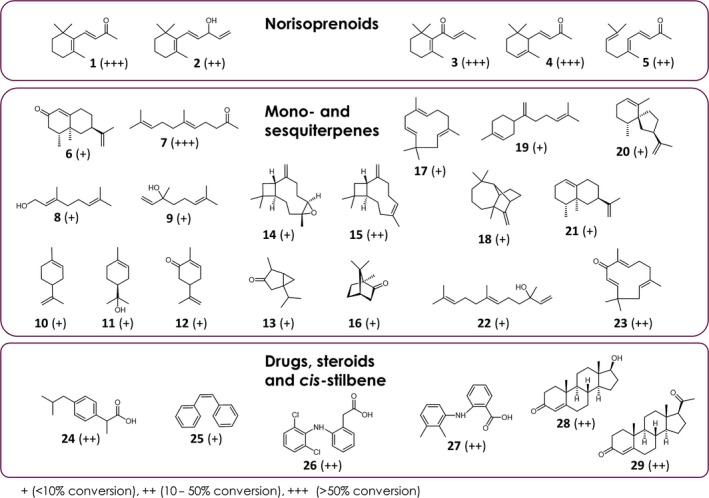
Substrate spectrum of CYP109Q5 from *C. apiculatus* DSM436. *In vitro* biotransformations were performed with purified P450 (2 μM) and CamA and CamB as the redox partners for 24 h. Conversions were estimated based on the product areas relative to the internal standard carvone. 1: β‐ionone; 2: vinylionol; 3: β‐damascone; 4: α‐ionone; 5: pseudoionone; 6: nootkatone; 7: geranylacetone; 8: nerol; 9: linalool; 10: limonene; 11: α‐terpineol; 12: carvone; 13: thujene; 14: β‐caryophylleneoxide; 15: β‐caryophyllene; 16: camphor; 17: α‐humulene; 18: longifolene; 19: β‐bisabolene; 20: premnaspirodiene; 21: valencene; 22: nerolidol; 23: zerumbone; 24: ibuprofen; 25: *cis*‐stilbene; 26: diclofenac; 27: mefenamic acid; 28: testosterone; 29: progesterone.

First tests were carried out *in vitro* using three purified heterologous redox partner systems, CamA and CamB from *Pseudomonas putida*, FdR and FdX from *E. coli* JM109, or FdR in combination with AdX (*bos taurus*) to increase the probability of successful activity reconstitution. Since the already characterized CYP109 members are able to oxyfunctionalize β‐ionone, valencene and fatty acids, we focused on these substrates to find a functional combination with the redox partners. Interestingly, β‐ionone and valencene were hydroxylated when electrons were delivered by CamA/CamB from *P. putida*, while only slight or no activity was observed with the combinations FdR/FdX and FdR/AdX respectively. Therefore, CamA/CamB was used to investigate the substrate spectrum of CYP109Q5 (Fig. 1). In addition to different norisoprenoids, both, cyclic and acyclic monoterpenes, such as geranylacetone or limonene were hydroxylated by the enzyme. In addition, CYP109Q5 was able to hydroxylate *cis*‐stilbene and ibuprofen, as well as a variety of sesquiterpenes such as α‐humulene or β‐bisabolene. CYP109Q5 also exhibited activities towards diclofenac, mefenamic acid, nerolidol, camphor as well as the steroids progesterone and testosterone. The hydroxylation of fatty acids as characteristic of the CYP109 family was only achieved in traces with CYP109Q5 (Table S2). Overall, CYP109Q5 has thus shown to accept a broad spectrum of substrates with different functionalities and size.

### Structure of CYP109Q5

**Figure 2:**
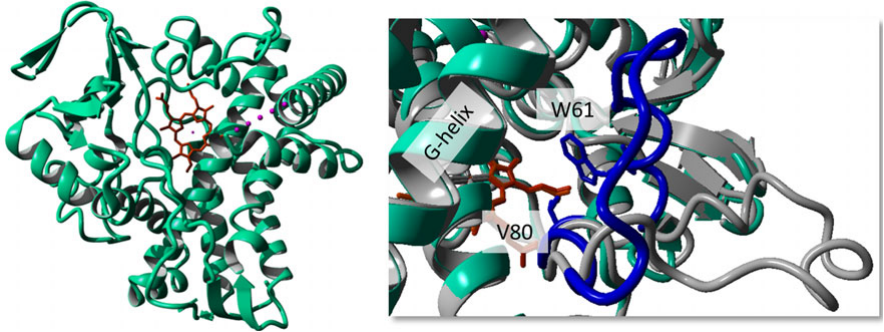
Crystal structure of CYP109Q5. The monomer as well as a comparison with the vitamin D3 25‐hydroxylase from *Pseudonocardia autotrophica* (PDB code 5GNL,* grey*) is shown with the haem in *orange*. The main difference in both structures is the movement of the N58‐P77 loop in CYP109Q5 (*blue*) bringing W61 and V80 to the substrate access channel.

In order to prepare protein for X‐ray crystallographic studies, the gene encoding CYP109Q5 was codon‐optimized for expression in *E. coli* and purchased from GenScript already ligated into pET‐28a(+), equipping the protein with an N‐terminal histidine tag. The gene was expressed in *E. coli* BL21(DE3) and purified using nickel affinity and gel filtration chromatography. Crystals of CYP109Q5 diffracted to 1.55 Å and were in the *P*2_1_1 space group, with one monomer in the asymmetric unit. Data collection and refinement statistics can be found in Table S3. The polypeptide chain was complete except for a region G165 to A175, constituting the ‘FG loop’, which could not be modelled presumably to its mobility (Fig. 2). The structure of CYP109Q5 was observed to adopt the classical P450 fold, and analysis using the DALI server (Holm and Laakso, 2016) suggested that the closest structural homologue was a vitamin D3 25‐hydroxylase from *Pseudonocardia autotrophica* (PDB code 5GNL), with 36% sequence identity, and giving an rmsd of 1.5 Å over 362 C‐α atoms. The major difference between the two enzyme structures was an extended loop between N58 and P77, which, in CYP109Q5, has folded up to meet the G‐helix, possibly restricting access to the haem (Fig. 2). The active site cavity is large and hydrophobic, with I232 and A236 at one side of the haem, and W61 and V80 brought to the other side of the substrate access channel by the movement of the N58‐P77 loop. V80 corresponds to F87 in P450 BM3, which plays a crucial role in the determination of activity and selectivity (Whitehouse *et al*., 2012). However, the position of W61, whose indole N atom is in contact with the haem propionate, is not a core element in the P450 superfamily and is thus special to the CYP109Q5 structure. The residue A280 is also part of the active site that is the fifth amino acid after the conserved ExxR motif the alteration of which also showed great influence on the enzyme properties (Seifert and Pleiss, 2009). In addition, a big cavity could be seen in the surface display which presumably enables the accommodation of bulky and/or long acyclic substrates (Fig. S3). Finally, the high‐resolution structure enabled us to precisely alter the enzyme properties by rational enzyme engineering.

### Rational enzyme engineering of CYP109Q5

**Figure 3:**
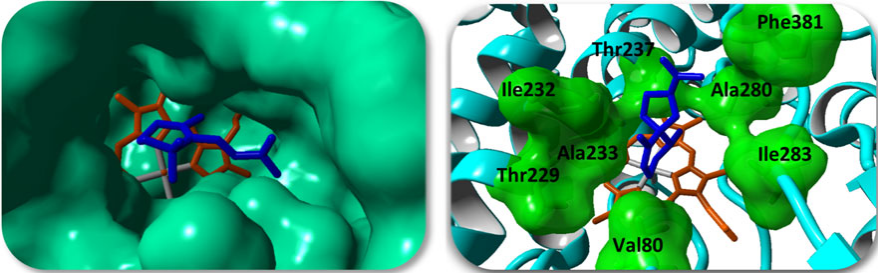
Strategy of the rational enzyme design of CYP109Q5. The substrates β‐ionone (*blue, left*) and premnaspirodiene (*blue, right*) were modelled in the active pocket using the AutoDock Vina algorithm implemented in YASARA. *Right*: The surfaces of the amino acids for mutagenesis are shown in green which were selected based on literature data (3DM‐database) and contacts to the ligands.

By the use of CYP109Q5 from *C. apiculatus* DSM436, a diverse set of substrates such as terpenes as well as many other important compounds could be converted. More interestingly, besides the allylic 4‐hydroxy‐β‐ionone, two further products with *m/z* of 208 corresponding to putative hydroxylation or epoxidation products were observed in the biotransformation with β‐ionone as substrate. At this point, the approach of rational enzyme engineering was of great interest to influence the activity and selectivity positively for enabling determination of the exact structures. Furthermore, we also wanted to determine whether we can influence oxyfunctionalization of the sesquiterpenes. For this purpose, the crystal structure of CYP109Q5 was analysed using a P450‐3DM database, and additionally, β‐ionone, as well as premnaspirodiene, was modelled into the active site using AutoDock VINA implemented in YASARA Structure (Trott and Olson, 2010). The result exhibiting the highest binding energy of 8.50 kcal mol^−1^ for β‐ionone and 7.93 kcal mol^−1^ for premnaspirodiene is shown in Fig. 3. Based on the analysis on hotspot regions in P450 enzymes and the contact to the docked substrates using the database, amino acid residues were selected for semirational mutagenesis (Fig. 3, *right*). In particular, hydrophobic amino acids of different sizes were chosen for mutation. The database also provided information about which amino acid distribution exists in the entire P450 superfamily at a specific position. A focused alignment (subset) with terpene‐hydroxylating P450s was further used to determine amino acids occurring at the corresponding position. Besides V80, I232, A233 and A280, further amino acids close to the catalytically active haem were also addressed (T237, I283 and T229). In total, 28 single variants were finally generated by the QuikChange^®^ method using the pBAD33 construct carrying the native CYP109Q5 gene. All variants were heterologously expressed in *E. coli* strain JW5510 and tested for 24 h *in vitro* using cell lysate of the respective P450 variant with CamA/CamB as redox partners. The results with selected terpenes were analysed and the effect of the exchange determined based on the product peak areas relative to the internal standard (Table S4, Fig. S4). As already observed with the wild‐type enzyme, several products were detected over a reaction time of 24 h, the distribution of which sometimes differed significantly from that of the wild‐type. Overall, the respective variant showed similar effects such as an increased activity with the different terpenes. Substitutions with the most impact on activity and/or selectivity after mutagenesis were V80G/A, T229S/L, T237S, A280I/V and I283L. The substitution of position V80 resulted in a strong effect on activity and selectivity. While the small hydrophobic amino acids glycine and alanine caused partial increase in activity with most of the terpenes, the activity decreased considerably over leucine to isoleucine compared to the wild‐type (valine). The mutations T229L and T229S had a positive influence on activity and led to the largest change of product distribution in comparison with all variants. This could be observed especially with the norisoprenoids β‐ionone and vinylionol. In the P450 3DM‐superfamily (~38 000 sequences), 85% of the proteins exhibit a threonine at position 237, which is associated with O_2_ activation (Aikens and Sligar, 1994; Gober *et al*., 2016). Threonine was therefore substituted by serine (6% of proteins), which had a positive effect on activity and selectivity. Investigating the influence of hydrophobic residues of varying size, hotspot position A280 was substituted correspondingly. While mutating A280 to leucine and phenylalanine resulted in a negative effect on the activity of CYP109Q5, A280I and A280V showed a strong increase in activity with a concomitant change in product distribution with most of the substrates. A similar positive effect could be achieved with the mutagenesis of position I283, in particular with the variant I283L. Since these exchanges are mostly located on the opposite side of the active pocket (Fig. 3), we investigated the combinations of the most promising variants focusing on the positions V80 and A280, thereby generating ten double variants the effects of which were analysed similar to the single variants (Table S4). All the double variants, combining V80G or V80A and five replacements at position A280, had strong positive influences on the activity and selectivity of CYP109Q5. Furthermore, increased activities were achieved with the double variants V80G/I283L and V80G/T237S respectively. However, these variants showed low functional expression, characterized by a strong maximum at 420 nm in the CO difference spectrum (data not shown). For the double variants V80G/T229S and V80G/T229L, respectively, no actively folded P450 was obtained after expression. By contrast, the combination A280V/T229L could be well expressed and showed a strong positive effect on activity and selectivity compared to the wild‐type.

### *In vitro* characterization using partially purified enzyme

**Figure 4:**
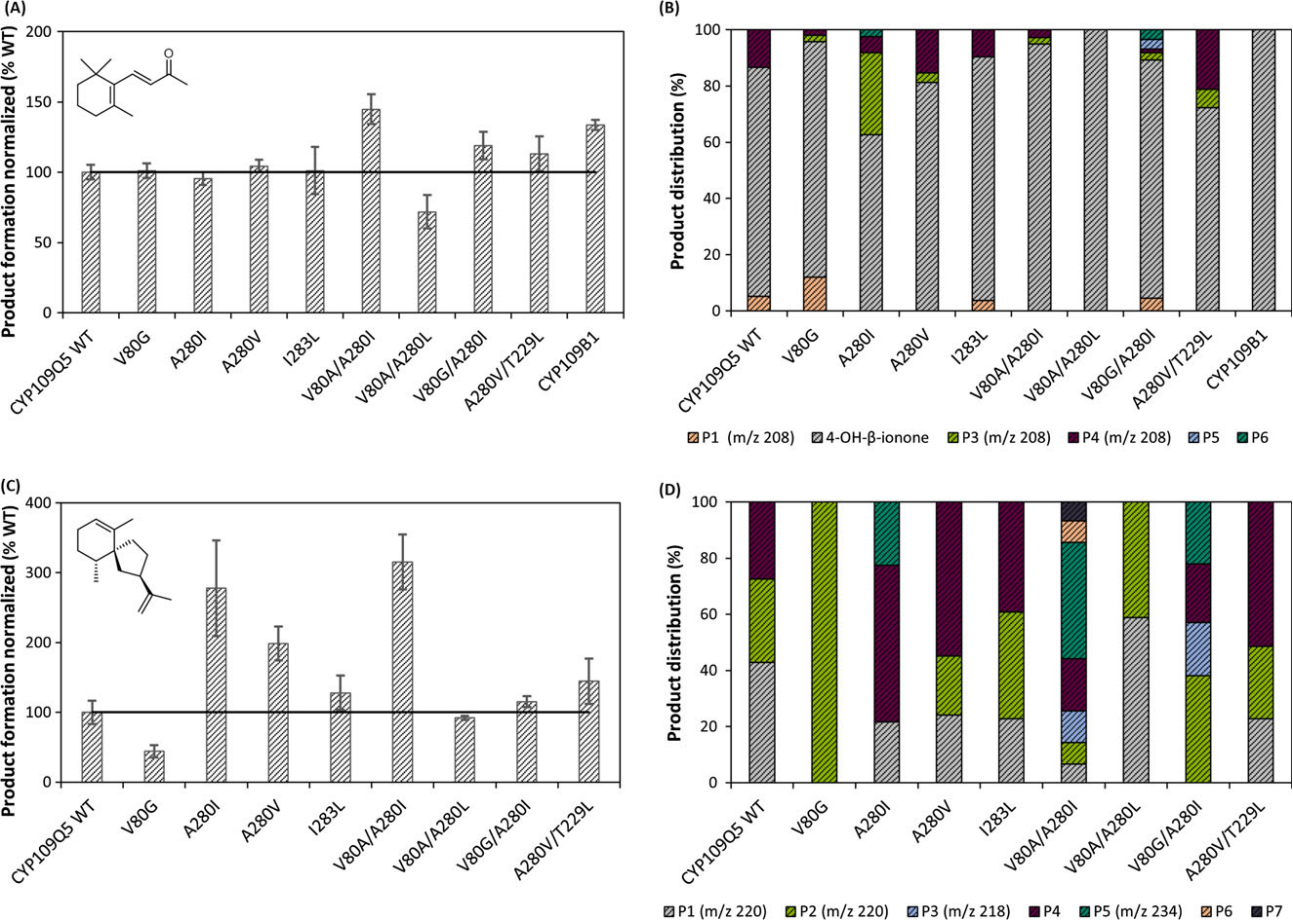
CYP109Q5 *in vitro* product formation and distribution using β‐ionone (A and C) and premnaspirodiene (B and D) as substrates. The CYP109Q5 wild‐type, selected variants and in case of β‐ionone CYP109B1 from *B. subtilis* were tested as purified enzymes with CamA and CamB as redox partners. Biotransformations were performed with 2 μM purified P450 and 1 mM substrate for 2 h and analysed by GC‐FID and GC‐MS. For the quantification of the products, 0.2 mM carvone was used as internal standard. A threshold value of 0.07 (area product divided by area of internal standard) was defined for each product and total product formation was normalized on the wild‐type CYP109Q5. Following values of area product divided by area internal standard were reached with the purified wild‐type enzyme: 4.25 (β‐ionone); 0.44 (premnaspirodiene).

For detailed *in vitro* characterization, eight variants showing the greatest effects in terms of activity and selectivity compared to the wild‐type were selected. In total, four single variants (V80, A280I, A280V and I283L) and four double variants (V80A/A280I, V80A/A280L, V80G/A280I and A280V/T229L) were successfully partially purified by anion exchange chromatography and their concentration and functionality determined by CO difference spectroscopy (Table S5). The biotransformations with the partially purified variants and the wild‐type were carried out for 2 h *in vitro* with CamA/CamB as redox partners with selected substrates (Fig. 4 and S4). For comparison, CYP109B1 from *B. subtilis* was also used, because the products of the biotransformations using this enzyme with β‐ionone and valencene as substrates are known (Girhard *et al*., 2010). Although purification and reduction of the reaction time reduced the by‐product formation to a small extent, many different products and other oxidations were detected, which were finally analysed by GC‐FID and GC‐MS. Using β‐ionone as a substrate, similar activities were achieved with the different variants (Fig. 4). The wild‐type formed 0.75 ± 0.04 mM of hydroxylated products using 1 mM of β‐ionone after 2 h reaction time. Interestingly, with some variants, shifts in product distribution from the allylic product towards two further oxyfunctionalized products (*m/z* 208) were obtained confirming the previous lysate screenings. Using the variants, products such as P3 (A280I) as well as shifts to the product P4 (A280V/T229L) could be achieved. Similar shifts were observed with the other norisoprenoids such as vinylionol or β‐damascone as substrates (Fig. S5). With the substrate premnaspirodiene, an approximately threefold increase in activity could be obtained with the variants A280I and V80A/A280I compared to the wild‐type. Both variants also catalysed further oxidation to multiple oxyfunctionalized products (*m/z* 234). With the variants, the ratios of P2, P4 and P5 shifted rather than that new products were formed. The greatest increase in activity was measured with valencene as substrate (Fig. S5). An increase of up to ten times was achieved with variant V80A/A280I, which mainly showed further oxidation products of nootkatone. In biotransformations with possible products such as nootkatone and caryophyllene oxide, activity was generally higher than that of the similar non‐oxyfunctionalized compounds α‐humulene and valencene. In addition, other compounds such as diclofenac, mefenamic acid and aromadendrene were also tested and in some cases, high increase in activity, especially with the V80A/A280I variant, was observed (Fig. S5). With diclofenac, ibuprofen and mefenamic acid, respectively, 4′‐hydroxydiclofenac, 2‐hydroxyibuprofen and 3′‐hydroxymethylmefenamic acid, respectively, were selectively formed using all variants. In total, high impact on selectivity and/or activity of the studied variants could be demonstrated compared to the CYP109Q5 wild‐type.

### CYP109Q5 whole‐cell biocatalyst

Since for most of the terpenes no product standards are available, the identification of the products turned out to be difficult. First indications were obtained using GC‐MS analysis, but these do not allow the exact structure elucidation. In order to generate sufficient product titres for solving the structure of unknown products, we constructed a dual‐vector system consisting of pBAD33 carrying the P450 and the compatible pBAD18 vector including CamA/CamB, each under the same arabinose‐inducible promoter system in *E. coli* JW5510. The best result with the highest activity per cell was finally obtained by induction with 0.1% (*v*/*v*) L‐arabinose and expression at 25°C overnight (Fig. S6). The experiments with CYP109Q5 were performed *in vivo* after expression in the dual‐vector system with resting cells. High activities were achieved when β‐ionone from a DMSO stock was used as substrate. For characterization of the products, the preparative biotransformations were carried out with the CYP109Q5 variant A280V/T229L, which showed high activity *in vitro* with a simultaneous shift of the product spectrum towards the previously unknown hydroxylated products (Fig. 4). In total, 0.5 g l^−1^ of the oxyfunctionalized products were generated in the preparative biotransformations in 100 ml scale after 24 h. Semipreparative HPLC purification allowed the isolation of all three products efficiently for NMR analysis (Fig. S7). The main product (74%) was the allylically hydroxylated 4‐hydroxy‐β‐ionone. The second product peak (7%) in the GC‐FID or first in the semipreparative HPLC turned out to be 3‐hydroxy‐β‐ionone. Furthermore, 2‐hydroxy‐β‐ionone was identified as a third product. Also hydroxylated in non‐allylic position, it was formed as 19% proportion of the product mixture. In contrast to the *in vitro* reactions, the whole‐cell system finally produced sufficient amounts for the structure elucidation.

## Discussion

In this work, we focused on the identification and further development of new P450 biocatalysts for the oxyfunctionalization of different terpenes. While many examples exist for the allylic hydroxylation of β‐ionone and the hydroxylation of valencene, so far only a few P450s are known capable of accepting more bulky sesquiterpene candidates of the terpene spectrum (Janocha *et al*., 2015). Instead of specialists, we were looking for generalists who can be used in manyfold ways in the fragrance and flavour industry. Depending on future application, biocatalysts can finally be transformed into a specialist with specific properties by protein engineering (Bornscheuer *et al*., 2012; Otte and Hauer, 2015). P450s from a family or subfamily show often similar substrate spectra. For the identification of novel terpene hydroxylases, we therefore searched for organisms that possess large numbers of potentially interesting P450s for the oxyfunctionalization of terpenes. Studies on the myxobacterium *S. cellulosum* So ce56 have already identified and characterized P450s, able to efficiently hydroxylate norisoprenoids and sesquiterpenes (Ly *et al*., 2012; Schifrin *et al*., 2015; Litzenburger and Bernhardt, 2016). Myxobacteria feature the largest bacterial genomes known today and a versatile secondary metabolism, making them particularly interesting for biotechnological applications (Schneiker *et al*., 2007; Diez *et al*., 2012). With *C. apiculatus*, a related myxobacterium, the P450s of which have not yet been investigated, was selected. Use of the strictly regulated pBAD33 expression vector was the key factor for the production of CYP109Q5 from *C. apiculatus* DSM436. It turned out that the activity reconstitution was enabled using CamA/CamB as heterologous redox partners which are thus suitable to supply electrons to multiple P450s (Bell *et al*., 2010; Reisky *et al*., 2018). The enzyme displayed a wide range of catalysed reactions from diverse norisoprenoids, steroids, non‐steroidal anti‐inflammatory drugs to mono‐ and sesquiterpenes (Fig. 1). Interestingly, CYP109Q5 showed only minimal activity with fatty acids, which distinguishes this enzyme from the CYP109 members that are actually declared as fatty acid hydroxylases, such as CYP109B1, CYP109C1, CYP109C2 or CYP109D1 (Girhard *et al*., 2010; Khatri *et al*., 2013). The amino acid sequence identity of this novel *C. apiculatus* enzyme is 39% to CYP109B1, 44% to CYP109C1, 43% to CYP109C2 and 38% to CYP109D1. Studies on the steroid hydroxylating CYP109E1 from *B. megaterium* (36% identity to CYP109Q5) showed that this enzyme is more closely related to the steroid hydroxylase CYP106A1 in comparison with the already characterized CYP109 enzymes (Jóźwik *et al*., 2016). Classification into a P450 family thus represents a strong evidence for a particular substrate spectrum; this is reflected by CYP109B1 and CYP109E1 that also functionalize a broad spectrum of substrates including steroids, fatty acids and terpenes (Girhard *et al*., 2010; Putkaradze *et al*., 2017). However, the preferences or natural substrates may differ in large and heterogeneous P450 families such as the CYP109 family.

**Figure 5:**
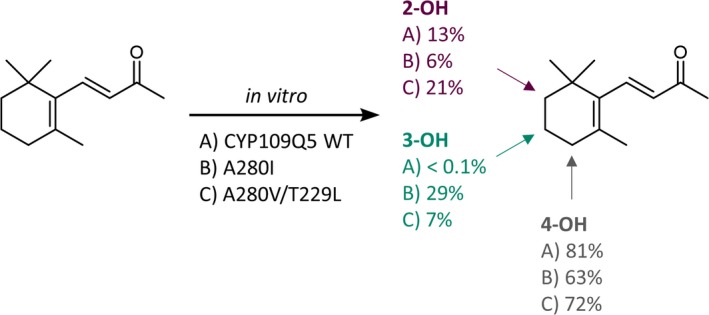
CYP109Q5‐catalysed oxyfunctionalization of β‐ionone. The *in vitro* product distribution is presented for the wild‐type and a selection of variants. The products 2‐hydroxy‐, 3‐hydroxy‐ and 4‐hydroxy‐β‐ionone were identified by NMR spectroscopy.

**Figure 6:**
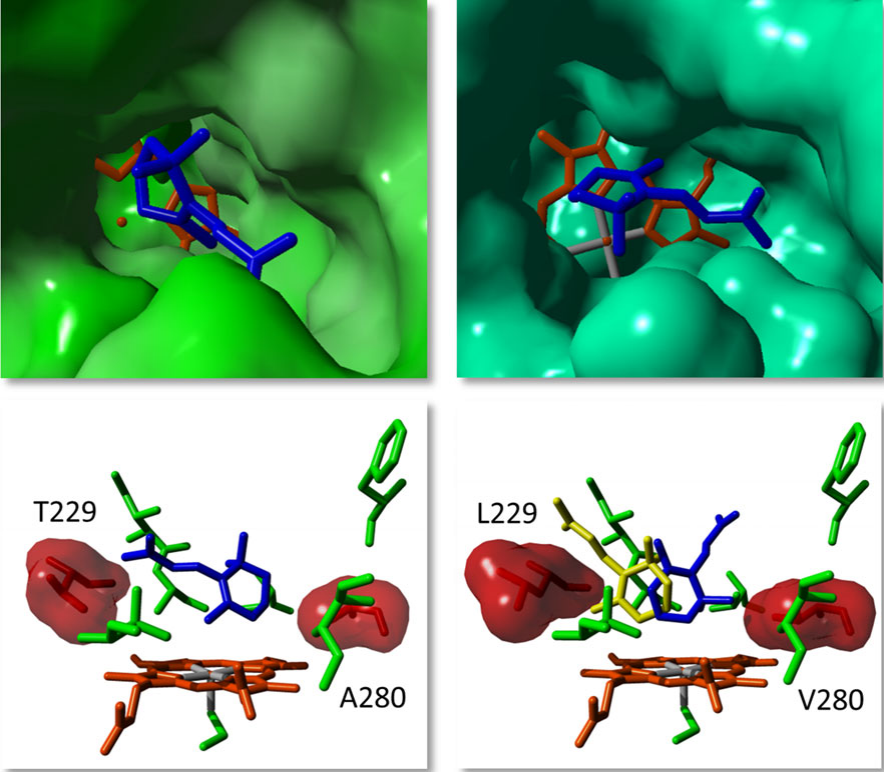
Comparison of active pockets of CYP101C1 from *N. aromaticivorans* and CYP109Q5 from *C. apiculatus*. In the case of the CYP109Q5 crystal structure, the substrate β‐ionone (*blue*) was bound into the active pocket using AutoDock Vina. *Top*,* left*: CYP101C1 cocrystallized with β‐ionone, PDB file: 3OFU *; Top*,* right*: crystal structure of CYP109Q5 wild‐type with β‐ionone*; Bottom*,* left*: lateral view of the active site of the wild‐type CYP109Q5; *Bottom*,* right*: lateral view of the active pocket of the CYP109Q5 variant A280V/T229L with the two docking results (*blue* and *yellow*) exhibiting the highest binding energy. *In silico* mutagenesis was performed by YASARA and the structure was subsequently energy minimized.

A combined strategy of substrate docking, comparison with other terpene hydroxylases, and mutational data from the literature was used for rational enzyme design. At the same time, the focus was set on two important positions in P450s (V80 and A280), which should have a decisive influence on activity and selectivity. In particular, the substitutions at positions V80, T229, I283 and A280 showed a large positive influence on the activity and changed the product spectrum, while partly even an additive effect was detectable. Position 57 in the 3DM database (V80 in CYP109Q5) as well as the position five amino acid residues after the conserved ExxR motif (A280) points towards the haem. It can be assumed that these residues interact with all substrates, resulting in a high influence on the activity and selectivity. The positions I283 and T229 are further away from the catalytically active haem iron, nevertheless also in direct contact with the substrates, presumably affecting selectivity. As crucial position for orienting the substrates for regioselective oxidation, T229 was identified as equivalent position to human CYP2A6 (N297; Di *et al*., 2009). Finally, eight variants were selected, partially purified and further characterized *in vitro* (Fig. 3 and S4). The reference CYP109B1 hydroxylated β‐ionone exclusively in the allylic position, while with the wild‐type and variants of CYP109Q5 two further hydroxylated by‐products could be identified. Hereby, the pBAD33/pBAD18 expression system proved to be extremely effective. The *in vivo* product formation of 0.5 g l^−1^ using CYP109Q5 A280V/T229L was far superior to that of similar systems, such as the P450s from *S*.* cellulosum* with titres in the lower mg l^−1^ range (Khatri *et al*., 2011a; Litzenburger and Bernhardt, 2016). Compared to the most active β‐ionone‐biocatalyst known today, CYP101B1 (0.83 g l^−1^), this is only a slightly lower titre (Hall and Bell, 2015). The ratio of the products was equivalent to that of the *in vitro* biotransformations. This finally allowed the purification and structure elucidation of the by‐products formed by the CYP109Q5 variant A280V/T229L. Interestingly, in addition to the allylic oxidized main product, these two main by‐products were found to be hydroxylated in non‐allylic positions of the ionone ring (Fig. 5). With CYP101B1 and C1 from *Novosphingobium aromaticivorans* and CYP264B1 from *S*.* cellulosum* as exceptions (Bell *et al*., 2010; Ly *et al*., 2012), P450s usually hydroxylate in an allylic position (Table S6). Even excessive mutational studies with P450 BM3 have failed to yield any variants showing activity in the non‐allylic position of norisoprenoids (Eiben *et al*., 2006; Urlacher *et al*., 2006; Venkataraman *et al*., 2012). The reason for this is the reactivity of the positions in the ionone ring. The regioselectivity is mainly controlled by the bond dissociation energy and the steric accessibility. The two methyl groups on the ionone ring direct the oxidation to the more sterically accessible C3. The electronic activation of the allylic C‐H bond (low dissociation energy) by the double bond on the ionone ring finally leads to an oxidation of the energetically favoured C4 position (Khursan *et al*., 1997; Gossauer, 2006; Janocha *et al*., 2015). The oxyfunctionalization of the C2 and C3 positions on the ionone ring by CYP109Q5 thus represents a specialty. For the first time, the functionalization of all three positions was achieved, the product distribution of which could be shifted especially with the variants A280I and A280V/T229L by mutagenesis. While the A280V/T229L double variant formed 21% 2‐hydroxy‐β‐ionone and 7% 3‐hydroxy‐β‐ionone *in vitro*, variant A280I produced 6% and 29% respectively (Fig. 5). A possible explanation for the observed activities and shifts can be found in the crystal structure of CYP109Q5 modelled with β‐ionone (Fig. 6). While CYP109Q5 has a very large active site pocket that can also accommodate steroids, for example, the crystal structure of CYP101C1 from *N. aromaticivorans* cocrystallized with β‐ionone reveals binding with low degree of freedom. This enzyme is able to hydroxylate β‐ionone in allylic and C3 position at a ratio of 75 to 25 (Bell *et al*., 2010). The activity can be rationalized by the crystal structure in which the C4 position of the ionone ring is closest to the active haem iron (Fig. 6, *top left*). In contrast, the docking experiments with CYP109Q5 indicate a possible orientation of the C2 and C3 positions of β‐ionone to the haem (Fig. 6, *top right*). Accordingly, there appears to be enough space in the active pocket for the functional bonding of all three positions, which may explain the formation of a mixture of all three hydroxylated products. Considering further the mutagenesis‐modified conformation of the A280V/T229L double variant, it can be seen that both positions have a decisive influence on the orientation of β‐ionone in front of the catalytically active haem (Fig. 6, *bottom left and right*). Similar product shifts were also observed with other norisoprenoids, which is why the production of non‐allylic oxyfunctionalized products is also likely here. The active pocket can be modelled accordingly to favour selectivity for a particular product and there are many more interesting positions like W61, combinations, positions in the substrate access channel or loops which might have a high impact on enzyme properties.

With respect to the other terpenes, some variants led *in vitro* to enormous increases in product formation (Fig. 4 and S4). At the same time, in comparison with the wild‐type, new or even multiple oxyfunctionalized products were formed using the most active variants. Initially, this gave the impression that the selectivity was reduced by mutagenesis. However, it was found that some products were increasingly formed due to higher activity compared to detected product traces with the wild‐type. Molecular masses of 220 or 234 also suggest alcohol overoxidation to the corresponding ketone or aldehyde presumably catalysed by the P450 *via* a *gem*‐diol; such activity has already been described in previous work (Sowden *et al*., 2005). In fact, the formed products seem to be better substrates than the original substrates, which can be explained, *inter alia*, by increased water solubility and reactivity through the first oxyfunctionalization. This is corroborated by a higher activity towards the potential product nootkatone (Fig. S5).

There are only few examples for biocatalysts with activity towards the tested sesquiterpenes such as α‐humulene, β‐bisabolene or premnaspirodiene, which makes the enzyme CYP109Q5 particularly interesting. The previous examples for the production of 5‐ or 8‐hydroxy‐α‐humulene were extended by the biocatalyst identified in this work (Yu *et al*., 2011; Schifrin *et al*., 2015). For the first time, oxyfunctionalization of β‐bisabolene was enabled by a heterologously expressed bacterial P450. Considering the hydroxylation of premnaspirodiene, so far only one example for a biocatalyst is known in the literature (Takahashi *et al*., 2007). Since multiple oxyfunctionalized products of premnaspirodiene with *m/z* 220 and 218 were obtained *in vitro* (Fig. 4, *down*), the formation of highly desired solavetivol and the further oxidized solavetivone (*m/z* 218) are likely. This is corroborated by the docking experiment showing the highest binding energy with a premnaspirodiene conformation that favours hydroxylation to solavetivol (Fig. 3, *right*). The *in vivo* generated products using CYP109Q5 are thus of particular interest for future applications.

Overall, CYP109Q5 has emerged as a universally applicable biocatalyst for the oxyfunctionalization of a broad range of norisoprenoids, sesquiterpenes and other compounds. Based on the developed whole‐cell system, production of industrially relevant products using this enzyme must be evaluated in future work. Furthermore, completely new products are conceivable the properties of which can be investigated by the CYP109Q5‐catalysed synthesis. Once these products have been identified, tailor‐made specialists can be designed based on the crystal structure and the outstanding evolvability of the enzyme. The ability to oxidize non‐activated C‐H bonds, as in the case of β‐ionone, makes CYP109Q5 particularly interesting for industrial processes and provides an excellent starting point for further investigation. Since even very large and bulky substrates such as progesterone are effectively oxyfunctionalized, evaluating di‐ and triterpenes as substrates is the next logical step which may enlarge the versatility of CYP109Q5 for future applications.

## Experimental procedures

### Cloning of CYP109Q5

The isolation of the genomic DNA from *Chondromyces apiculatus* DSM436 was carried out directly from a VY/2 agar plate. The cells were scraped off and resuspended in ddH_2_O. The recovery of the DNA was finally carried out with the Gene JET Genomic DNA Purification Kit (Thermo Fisher Scientific Inc., Waltham, MA, USA) and elution with 200 μl ddH_2_O. The gene of CYP109Q5 (EYF04172) was amplified by PCR using the KOD HS DNA polymerase (Novagene Inc., Madison, WI, USA). Appropriate primers were designed to introduce a *Nde*I as well as a *Hind*III restriction site on the 3′‐fragment end. Thereby, a more frequently used stop codon from *E. coli* was introduced. After PCR amplification, the fragment was subsequently subcloned into the pET‐28a(+) vector behind the poly‐histidine‐tag through the corresponding restriction sites. The gene was further subcloned into pCWori^+^ and pBAD33 using the *Nde*I and *Hind*III restriction sites. DNA sequencing (GATC Biotech AG, Konstanz, Germany) verified the correct constructs. The sequences of the primers and the PCR procedure are given in Tables S7 and S8.

### Expression and purification

The heterologous protein expression in *E. coli* was carried out in 400 ml of TB medium containing the corresponding antibiotic in 2 l baffled flasks. A 3 ml overnight culture from a fresh transformation plate was used for inoculation, and the cultivation was continued at 37°C and 100 rpm until a OD_600_ of 1.5–1.8 was reached. Subsequently, the cultures were slowly cooled to the expression temperature and induced as listed in Table S9 to start protein expression. The expression was prolonged for 15 h at 100 rpm. After harvesting the cells for 15 min at 6000 *g* and 4°C, the pellets were frozen at −20°C until further processing. Cells containing a protein with a poly‐histidine‐tag (CamA, CamB and FdR) were resuspended to 0.25 g_cww _ml^−1^ in cold immobilized metal affinity chromatography (IMAC) buffer (50 mM KPi pH 7.0, 150 mM NaCl, 10% glycerol). Cold ion exchange chromatography (IEX) buffer (A: 50 mM KPi pH 7.5, 0.5% glycerol or B: 50 mM Tris/HCl pH 8.2 (25°C), 0.5% glycerol) was used to resuspend cells to 0.25 g_cww _ml^−1^ which expressed proteins for the purification *via* IEX (CYP109Q5, AdX, FdX). 0.1 mM PMSF was freshly added and the cell suspensions were sonicated (Branson Sonifier 250 equipped with a microtip: 3 mm diameter; Danbury, CT, USA) for 4 min on ice (pulse: Output 3–5, Duty cycle: 35%). Cell lysate was obtained by centrifugation at 55 000 *g* for 30 min at 4°C, and the supernatant was subsequently filtered over a 0.45 μm membrane filter. The N‐terminally poly‐histidine‐tagged proteins were purified by IMAC. Therefore, the filtered cell lysate was loaded on a His GraviTrap Talon^®^ resin (1 ml column volume ≙ cv; GE Healthcare, Freiburg, Germany) pre‐equilibrated with 8 cv IMAC buffer. After binding to the cobalt packed column, non‐specifically bound proteins were eluted with 5 cv IMAC buffer (10 mM imidazole) followed by 5 cv buffer containing 20 mM imidazole. The target proteins were eluted through 8 cv IMAC buffer with 150 mM imidazole. After that, the eluates containing the desired proteins were dialysed against 5 l ice‐cold IMAC buffer overnight (max. 30 ml eluate) to ensure removal of imidazole. If needed, the purified proteins were further concentrated by Vivaspin20 columns (cut‐off 10 kDa; Sartorius, Goettingen, Germany). For IEX, the columns Toyopearl DEAE 650M XK16/40 30 ml cv and GigaCap DEAE 650M XK50/20 90 ml cv (both Tosoh Bioscience, Griesheim, Germany) were applied on a ÄKTApurifier 10 system equipped with an Frac‐950 fraction collector (GE Healthcare). After 1 cv equilibration (buffer B), the lysate containing CYP109Q5 was loaded onto the GigaCap DEAE 650M column and washed with 2 cv of buffer B. A two‐step gradient (70 mM NaCl for 1 cv, 300 mM NaCl for 1.75 cv) was used to elute the target protein. The supernatants containing AdX and FdX in IEX buffer A were loaded on the 30 ml DEAE 650M resin equilibrated with 3 cv IEX buffer A. The column was washed with 2 cv of the same buffer before elution of bound proteins with a linear gradient of 0–1 M NaCl developed over 10 cv. Elution was followed by UV using A_420_ for CYP109Q5 or the specific absorption maxima of the redox proteins shown below. Fractions with the highest target protein to A_280_ ratio were combined and the buffer exchanged through Vivaspin20 columns (cut‐off 10 kDa) to the IMAC buffer while concentrating the samples. Finally, the IMAC and IEX purified proteins were aliquoted to 200 μl and stored at −20°C until use. The P450 concentrations were determined by CO difference spectroscopy according to the method of Omura and Sato using ε_450‐490_ = 91 mM^−1^ cm^−1^ (1964). Protein concentration of the redox partners was determined as follows: CamA (average of 378, 454 and 480 nm; (ε) 9.7, 10.0 and 8.5 mM^−1 ^cm^−1^); CamB (average of 415 and 455 nm; (ε) 11.1 and 10.4 mM^−1^ cm^−1^); FdR (456 nm; (ε) 7.1 mM^−1^ cm^−1^); FdX (464 nm; (ε) 8.42 mM^−1^ cm^−1^); AdX (414 nm; (ε) 11.0 mM^−1^ cm^−1^; Huang and Kimura, 1973; Fujii and Huennekens, 1974; McIver *et al*., 1998; Purdy *et al*., 2004).

### Crystallization of CYP109Q5

A gene encoding CYP109Q5, codon‐optimized for expression in *E. coli*, was purchased from GenScript, already ligated into a pET‐28a(+) plasmid. The construct contained an N‐terminal histidine tag to achieve higher purities for crystallization. Following transformation of *E. coli* BL21(DE3) cells, a single colony was used to inoculate a 10 ml culture in LB broth containing 30 μg ml^−1^ kanamycin, which was grown overnight with shaking at 37°C. 10 ml of the overnight culture was then used to inoculate 500 ml of TB containing 30 μg ml^−1^ kanamycin. This larger culture was grown at 37°C with shaking until the OD_600_ reached a value of 1.0. The culture was then induced with 0.4 mM IPTG and supplemented with 0.2 mM FeSO_4_ and 0.5 mM 5‐Ala. Expression was conducted overnight at 18°C with shaking. Cells were harvested by centrifugation at 5000 *g* for 30 min. The pellets were resuspended in a buffer containing 50 mM K_2_HPO_4_, 300 mM KCl and 30 mM imidazole at pH 7.0, with 0.1 mM PMSF. The cells were ruptured using a French press at 40 kPsi. The lysate was centrifuged at 50 000 *g* for 45 min at 4°C. The soluble fraction was loaded onto a HisTrap FF column (GE Healthcare) pre‐equilibrated with the buffer and eluted with an increasing gradient of imidazole. Fractions were analysed by SDS‐PAGE and those deemed to contain CYP109Q5 were pooled, concentrated to a volume of 500 μl, and loaded onto a size exclusion column (Hiload 16/60 Superdex 200 PrepGrade; GE Healthcare) pre‐equilibrated with a buffer containing 50 mM Tris and 300 mM NaCl at pH 7.5. Fractions were again analysed by SDS‐PAGE and those deemed to contain CYP109Q5 of the best purity were pooled and concentrated using Vivaspin20 columns (cut‐off 10 kDa) for crystallization trials (Fig. S8).

CYP109Q5 was crystallized using high‐purity protein samples of concentration equal to 15 mg ml^−1^. INDEX and PACT screens were set up using a Mosquito^®^ robot (TTP Labtech, Melbourn, UK) in MRC plates using sitting drop method. CYP109Q5 crystals were grown in conditions containing 0.2 M NaCl, 0.1 M Tris pH 8.5 and 28% (w/v) PEG3350. The most promising crystals were flash‐cooled in liquid nitrogen using 10% glycerol in the mother liquor as cryoprotectant, and retained for analysis at the synchrotron.

### Data collection and refinement

A complete data set for CYP109Q5 was collected at the Diamond Light Source, Didcot, Oxfordshire, U.K., on beamline I04. The data were processed and integrated using XDS (Kabsch, 2010) and scaled using SCALA (Evans, 2006) within the Xia2 processing software (Winter, 2010). Data collection statistics are given in Table S3. Crystals of CYP109Q5 were in space group *P*2_1_1. The structure was solved using MOLREP (Vagin and Teplyakov, 1997), using CYP109A2 (5OFQ, Abdulmughni *et al*., 2017) from *Bacillus megaterium* as the search model. The solution contained one monomer in the asymmetric unit and the solvent content was 42.4%. The structure was built and refined using iterative cycles using Coot (Emsley and Cowtan, 2004) and REFMAC (Murshudov *et al*., 1997), employing local NCS restraints. The final structure had *R*_cryst_ and *R*_free_ values of 18.5% and 22.0%. Refinement statistics are presented in Table S3. The Ramachandran plot showed 97.3% of residues in allowed regions, 2.4% in preferred regions and 0.3% in outlier regions. Coordinate files and structure factors have been deposited in the Protein Data Bank (PDB) with the coordinate accession number 6GMF.

### Molecular docking and rational design

Molecular docking of β‐ionone and premnaspirodiene into the P450 active pocket was performed using the AutoDock Vina algorithm implemented in YASARA (Trott and Olson, 2010). The water molecules inside the active site and the channel were removed before for the docking experiment. *In silico* mutagenesis were applied by the swap tool in YASARA and subsequent energy minimization of the protein structure. Structural alignment with 5GNL was performed using the YASARA MUSTANG module. The rational design was conducted by site‐directed mutagenesis using the QuikChange^®^ method as described in the Supporting information (Table S10).

### In vitro biotransformations

The *in vitro* reactions were carried out in a volume of 200 μl in reaction buffer (50 mM KPi pH 7.0, 150 mM NaCl, 2.5% glycerol). The glucose 6‐phosphate dehydrogenase system (G6PDH from *L. mesenteroides*) was used to regenerate the cofactor NAD(P)H (12 U ml^−1^ G6PDH, 5 mM G6P, 1 mM MgCl_2_). Standard *in vitro* reactions contained 100 μg ml^−1^ catalase, 2 mM NAD(P)H and 1 mM substrate (50 mM stock solution in DMSO or ethanol, all tested substrates are listed in Table S2). All components were mixed, and the biotransformation was started with the addition of a mixture of P450 (lysate or purified), reductase, flavodoxin or ferredoxin in the stoichiometric ratio 1:1.5:15 (2 μM:3 μM:30 μM). The reactions were incubated for 2–24 h at 30°C and 180 rpm and subsequently prepared for analysis. Negative controls were either lysates generated by the expression of the empty vector, or in the case of reactions with partially purified P450s, all reaction components except the P450.

### Construction of a whole‐cell system

The dual‐vector system was created based on the compatible pBAD18 and pBAD33 vectors. The plasmid pBAD18_CamB_CamA was constructed *via* Gibson Assembly^®^ using the specific primers listed in Table S7. The fragments (vectors and inserts) amplified through PCR (Table S11) were controlled by an agarose gel and isolated using the Zymoclean™ Gel DNA Recovery Kit (Zymo Research Corp., Irvine, CA, USA) for purification. Gibson Assembly® was finally performed with 15 μl Gibson Assembly® mix and 5 μl DNA mix [vector to insert(s), molar ratio 1:5, total ~300 ng] for 1 h at 50°C. After transformation in chemically competent *E. coli* XL1‐blue cells, isolated plasmid constructs were checked for correct modification by DNA sequencing. For expression, the pBAD18 plasmid encoding the redox partners was transformed in competent *E. coli* JW5510 cells and new chemical competent cells prepared thereof. As next step, pBAD33 encoding the CYP109Q5 gene was freshly transformed each time prior expression. Expression of the three proteins was conducted in 400 ml of TB medium containing the corresponding antibiotic in 2 l baffled flasks. A 3 ml overnight culture from a fresh transformation plate was used for inoculation and the cultivation was continued at 37°C and 100 rpm until a OD_600_ of 1.5–1.8 was reached. Subsequently, the cultures were slowly cooled to 25°C and induced with 0.1% L‐arabinose, 0.5 mM FeSO_4_ and 0.5 mM 5‐Ala to start protein expression. The expression was prolonged for 15 h at 100 rpm. After protein expression, the cells were harvested for 15 min at 6000 *g* and 4°C and the resulting pellets directly resuspended with 100 mM KPi pH 7.0 supplemented with 30 mM glucose (to 0.2 g_cww _ml^−1^).

### Preparative in vivo biotransformations

The resting cell reactions were performed in 500 ml baffled shaking flasks with a reaction volume of 100 ml, a final cell concentration of 0.1 g_cww_ ml^−1^, with 30 mM glucose and with 5 mM β‐ionone (250 mM stock solution in DMSO) for 24 h at 30°C and 180 rpm. A 750 μl sample was taken of each reaction for GC analysis. The products were purified using reverse phase semipreparative HPLC and structurally elucidated by ^1^H‐, ^1^H‐COSY‐ and ^13^C‐NMR spectra as described in the Supporting information (Figs. S9–S11).

### Product analysis

The *in vitro* (200 μl) or *in vivo* (supernatant: 750 μl) samples were extracted with the same volume MTBE containing 0.2 mM carvone as internal standard. In case of a necessary derivatization of the analytes, acidification of the samples by 10 μl (*in vitro*) was performed before these were extracted with MTBE (including 0.3 mM 3‐methoxyphenylacetic acid as internal standard). Before derivatization, the organic phase was evaporated on a Genevac EZ‐2 Plus Evaporator (SP Scientific, Ipswich, UK) to dryness and the residual dissolved in a 90 μl mixture of 50% MTBE and 50% BSTFA + TMCS (99:1). Derivatization of the samples was carried out in GC vials for 30 min at 70°C. Gas chromatography was performed on a Shimadzu GC‐2010 equipped with an AOC‐20i autoinjector (Shimadzu, Nakagyo‐ku, Japan). The samples were injected with different split ratios (injector temperature 250°C, carrier gas H2, 30 cm s^−1^) and separated *via* a DB‐5 or HP‐1 ms‐ui column (both 30 m × 0.25 mm × 0.25 μm; Agilent Technologies Inc., Santa Clara, CA, USA). The analytes were detected by means of a flame ionization detector (FID, detector temperature 330°C) using the programs listed in Table S12. For initial product identification, the samples were afterwards analysed on a Shimadzu GCMS‐QP2010 detector using the identical temperature programs. In case of the non‐steroidal anti‐inflammatory drugs, product standards were available from a previous study using microbial biocatalysts (Klenk *et al*., 2018). HPLC measurements of diclofenac were performed as described elsewhere (Klenk *et al*., 2017). An Agilent 1260 HPLC (Agilent) equipped with single quad ESI‐MS was used for the LCMS analysis of the steroids testosterone and progesterone. The chromatographic separation of 10 μl sample was ensured with a Supelco C_18_ discovery column (150 × 4.0 mm, 5 μm; from Sigma‐Aldrich, St Louis, MO, USA). An isocratic gradient of 60% MeOH and 40% H_2_O + 0.1% v/v formic acid was used for 20 min at a flow rate of 1 ml min^−1^ at 30°C and a detection wavelength of 244 nm. MS parameters: ESI ionization; scan *m/z* 80–400; drying gas temperature 350°C; drying gas flow 9.0 l min^−1^; nebulizer pressure 35 psi; Vcap +3000 V; fragmentation voltage 70 eV. The sample preparation was performed by extraction with the same volume of MTBE (200 μl), evaporation of the organic phase on a Genevac EZ‐2 Plus Evaporator and uptake of the residues in 150 μl of the mobile phase. Examples of GC, HPLC and LCMS chromatograms are given in Figures S12–S16.

## Conflict of interest

None declared.

## Supporting information


**Fig. S1**. IEX purification of CYP109Q5.
**Fig. S2**. SDS‐PAGE und CO difference spectra of the purified CYP109Q5 wild‐type.
**Fig. S3**. Crystal structure of CYP109Q5.
**Fig. S4**. Initial screening of the CYP109Q5 single variants (lysate) with different terpenes.
**Fig. S5**. *In vitro* screening of the purified CYP109Q5 variants and/or CYP109B1.
**Fig. S6**. Investigation of L‐arabinose concentration and temperature for the expression of the dual‐vector system CYP109Q5 CamA/B.
**Fig. S7**. Semi‐preparative HPLC purification of the CYP109Q5 A280V/T229L preparative biotransformation with β‐ionone.
**Fig. S8**. SDS‐PAGE analysis of size exclusion chromatography of CYP109Q5 expressed using the codon‐optimized gene in the pET‐28a(+) vector.
**Fig. S9**. ^1^H and ^13^C‐NMR spectra of 3‐hydroxy‐β‐ionone.
**Fig. S10**. ^1^H and ^13^C‐NMR spectra of 4‐hydroxy‐β‐ionone.
**Fig. S11**. ^1^H and ^13^C‐NMR spectra of 2‐hydroxy‐β‐ionone.
**Fig. S12**. GC‐chromatogram of β‐caryophyllene *in vitro* biotransformation.
**Fig. S13**. GC‐chromatogram of β‐damascone *in vitro* biotransformation.
**Fig. S14**. GC‐chromatogram of lauric acid *in vitro* biotransformation.
**Fig. S15**. HPLC‐chromatogram of diclofenac *in vitro* biotransformation.
**Fig. S16**. LCMS‐chromatogram of testosterone *in vitro* biotransformation.
**Table S1**. Relationship of CYP109Q5 from *Chondromyces apiculatus* DSM436.
**Table S2**. List of substrates used in this study and overview of the results obtained with CYP109Q5.
**Table S3**. Data Collection and Refinement Statistics for CYP109Q5.
**Table S4**. Results of the rational enzyme design of CYP109Q5.
**Table S5**. CO difference spectra of the IEX purified CYP109Q5 variants.
**Table S6**. Biocatalysts with activity towards the investigated norisoprenoid β‐ionone.
**Table S7**. Primers used in this study.
**Table S8**. Reaction batches for the amplification from *Chondromyces apiculatus* genomic DNA.
**Table S9**. Expression conditions for the applied redox proteins within this study.
**Table S10**. QuikChange^®^ PCR approaches.
**Table S11**. Gibson Assembly^®^ approach.
**Table S12**. GC programs used in this study.
**Appendix S1**. Material and Methods.
