# Supplementary material for: Characterization and structure‐guided engineering of the novel versatile terpene monooxygenase CYP109Q5 from Chondromyces apiculatus DSM436
Source: Microb Biotechnol. 2018 Dec 27;12(2):377–91. doi: 10.1111/1751-7915.13354 (PMC6389848; doi:10.1111/1751-7915.13354)
Supplement: Supplementary file 1 — Fig. S1. IEX purification of CYP109Q5. Fig. S2. SDS‐PAGE und CO difference spectra of the purified CYP109Q5 wild‐type. Fig. S3. Crystal structure of CYP109Q5. Fig. S4. Initial screening of the CYP109Q5 single variants (lysate) with different terpenes. Fig. S5. In vitro screening of the purified CYP109Q5 variants and/or CYP109B1. Fig. S6. Investigation of L‐arabinose concentration and temperature for the expression of the dual‐vector system CYP109Q5 CamA/B. Fig. S7. Semi‐preparative HPLC purification of the CYP109Q5 A280V/T229L preparative biotransformation with β‐ionone. Fig. S8. SDS‐PAGE analysis of size exclusion chromatography of CYP109Q5 expressed using the codon‐optimized gene in the pET‐28a(+) vector. Fig. S9. 1H and 13C‐NMR spectra of 3‐hydroxy‐β‐ionone. Fig. S10. 1H and 13C‐NMR spectra of 4‐hydroxy‐β‐ionone. Fig. S11. 1H and 13C‐NMR spectra of 2‐hydroxy‐β‐ionone. Fig. S12. GC‐chromatogram of β‐caryophyllene in vitro biotransformation. Fig. S13. GC‐chromatogram of β‐damascone in vitro biotransformation. Fig. S14. GC‐chromatogram of lauric acid in vitro biotransformation. Fig. S15. HPLC‐chromatogram of diclofenac in vitro biotransformation. Fig. S16. LCMS‐chromatogram of testosterone in vitro biotransformation. Table S1. Relationship of CYP109Q5 from Chondromyces apiculatus DSM436. Table S2. List of substrates used in this study and overview of the results obtained with CYP109Q5. Table S3. Data Collection and Refinement Statistics for CYP109Q5. Table S4. Results of the rational enzyme design of CYP109Q5. Table S5. CO difference spectra of the IEX purified CYP109Q5 variants. Table S6. Biocatalysts with activity towards the investigated norisoprenoid β‐ionone. Table S7. Primers used in this study. Table S8. Reaction batches for the amplification from Chondromyces apiculatus genomic DNA. Table S9. Expression conditions for the applied redox proteins within this study. Table S10. QuikChange® PCR approaches. Table S11. Gibson Assembly® approach. Table S12. GC progr [file MBT2-12-377-s001.docx]

**Characterization and structure-guided engineering of the novel versatile terpene monooxygenase CYP109Q5 from *Chondromyces apiculatus* DSM436**

Jan M. Klenk,^1^ Paulina Dubiel,^2^ Mahima Sharma,^2^ Gideon Grogan,^2^ and Bernhard Hauer ^1^*

^1^ Institute of Biochemistry and Technical Biochemistry, University of Stuttgart, Germany

^2^ Department of Chemistry, University of York, Heslington, York, YO10 5DD, United Kingdom

**Supporting Information**

**Apendix 1. Material and Methods**

*Rational design*

The targeted base exchange was achieved by site-directed mutagenesis using the QuikChange^®^ method and the pBAD33 construct carrying the native CYP109Q5 gene. Due to the very high GC content of CYP109Q5 (template 70%, primer up to 78%), an increase in the DMSO concentration in the batch, which reduces the formation of secondary structures of the template and thus improves primer annealing (Chakrabarti and Schutt, 2001), was necessary to prevent insertion of primer multimers. The QuikChange^®^ approaches of 50 μL and the associated PCR programs are listed in the supplementary information (Table S8 and S10). After the PCR reaction, 1 μL of *Dpn*I (10 U μL^‑1^) was added and incubated at 37°C for 2 h to digest the methylated template DNA. A total of 8 μL QuikChange^®^ approach was used for transformation into 100 μL of chemically competent *E. coli* XL1‑Blue cells. Successful introduction of the desired mutation was confirmed after isolation of the plasmids by DNA sequencing.

*Product purification and identification*

For product purification, the supernatant of 4 x 100 mL from preparative biotransformations were extracted twice with the identical volume of MTBE. The organic phases were combined, evaporated to dryness and the residues were dissolved in 15 mL of acetonitrile and stored at 4°C for further use. The batches were finally purified by reverse phase semi-preparative HPLC on an Agilent 1200 System (St. Clara, California, USA) equipped with a G1311A quaternary pump, a HIP AS G1367B autosampler (1200 μL loop), a G1315D DAD detector and an analytical G1364C fraction collector. The separation of the analytes (injection volume: 1000 µL) was ensured using a Trentec Reprosil® 100-5 C18 column (250 × 20 mm, 5 μm) from Dr. Maisch GmbH (Ammerbuch-Entringen, Germany). The mobile phases A and B were composed of water containing 0.1% formic acid and acetonitrile, respectively. Elution was done in a gradient mode at a flow rate of 4.9 mL min^-1^ and a column temperature of 20°C using following program: 80% A (0 min), 55% A (65 min), 15% A (65.01 min), 15% A (90 min), 80% A (90.01 min), 80% A (105 min). The elution of the substrate and products were followed spectrophotometrically at a wavelength of 287 nm (Figure S5). Identical product fractions were combined, the solvent mixture of acetonitrile and water extracted three times with the same volume MTBE. Subsequently, the solvent was removed on a rotary evaporator and completely dried by a nitrogen gas flow. The remaining oil products were finally dissolved in *d*-chloroform and analyzed by ^1^H- and ^13^C-NMR to clarify the chemical structures. The purity of all samples were determined by GC. From 480 mg β-ionone 220 mg purified products were isolated (isolated yield: 42.3%).

*NMR measurements*

For the characterization of the purified products, NMR spectroscopy was used. ^1^H‑, ^1^H‑COSY‑ and ^13^C‑NMR spectra were recorded using a Bruker Avance 500 spectrometer at 500.15 and 125.76 MHz, respectively (Bruker, Billerica, Massachusetts, USA). The chemical shift *δ* was measured in ppm (parts per million) and referred to TMS (tetramethylsilane) *δ* = 0 ppm as a standard. 10 mg of the products were used for NMR analysis. The NMR spectra are given in the supplemental material (Figures S6-S8).

4-Hydroxy-β-ionone: ^1^H-NMR (500 MHz, CDCl_3_): *δ* (ppm) 1.06 (s, 3H, C-11), 1.07 (s, 3H, C-12), 1.43 – 1.49 (m, 1H, C-2), 1.63 – 1.70 (m, 1H, C-2), 1.70 – 1.74 (m 1H, C-3), 1.85 (s, 3H, C-13), 1.90 – 1.98 (m, 1H, C-3), 2.32 (s, 3H, C-10), 4.04 (t, 1H, *J* = 4.9 Hz, C-4), 6.13 (d, 1H, *J* = 8.1 Hz, C-8), 7.2 (d, 1H, *J* = 8.2 Hz, C-7).

^13^C-NMR (126 MHz, CDCl_3_): *δ* (ppm) 18.5 (C13), 27.3 (C10), 27.5 (C11), 28.2 (C3), 28.8 (C12), 34.6 (C1, C2), 70.0 (C4), 133.1 (C7), 133.8 (C5), 139.6 (C6), 143.0 (C8), 199.0 (C9).

3-Hydroxy-β-ionone: ^1^H-NMR (500 MHz, CDCl_3_): *δ* (ppm) 1.11 (s, 3H, C-11), 1.12 (s, 3H, C-12), 1.5 (t, 1H, *J* = 3.5 Hz, C-2), 1.77 (s, 3H, C-13), 1.80 – 1.84 (m, 1H, C-2), 2.06 – 2.11 (m, 1H, C-4), 2.30 (s, 3H, C-10), 2.41 – 2.47 (m, 1H, C-4), 3.98 - 4.04 (m, 1H, C-3), 6.12 (br d, 1H, *J* = 16.2 Hz, C-8), 7.21 (br d, 1H, *J* = 16.4 Hz, C-7).

^13^C-NMR (126 MHz, CDCl_3_): *δ* (ppm) 21.6 (C13), 27.3 (C10), 28.6 (C12), 30.0 (C11), 36.9 (C1), 42.7 (C2), 48.3 (C4), 64.6 (C3), 132.4 (C7), 132.7 (C5), 135.6 (C6), 142.5 (C8), 198.7 (C9).

2-Hydroxy-β-ionone: ^1^H-NMR (500 MHz, CDCl_3_): *δ* (ppm) 1.08 (s, 3H, C-11), 1.12 (s, 3H, C-12), 1.75 (s, 3H, C-13), 1.71 – 1.79 (m, 1H, C-3), 1.82 – 1.88 (m, 1H, C-3), 2.12 – 2.24 (m, 2H, C-4), 2.30 (s, 3H, C-10), 3.56 (dd, 1H, *J*= 9.1, 3.1 Hz, C-2), 6.11 (br d, 1H, *J* = 16.6 Hz, C-8), 7.21 (br d, 1H, *J* = 16.2 Hz, C-7).

^13^C-NMR (126 MHz, CDCl_3_): *δ* (ppm) 21.4 (C13), 22.1 (C10), 26.1 (C12), 26.7 (C11), 27.3 (C1), 30.5 (C4), 39.0 (C3), 75.4 (C2), 132.5 (C7), 134.2 (C5), 134.3 (C6), 142.9 (C8), 198.7 (C9).

Table S1. Relationship of CYP109Q5 from *Chondromyces apiculatus* DSM436.

|  |  | **Relationship** | | | |
| --- | --- | --- | --- | --- | --- |
| **P450 Name** | **Gene ID** | **Species** | **P450 Name** | **Sequence identity [%]** | **Characterized members** |
| CYP109Q5 | WP_044244829 | *Chrondromyces crocatus* | CYP109Q1 | 70 | CYP109B1(Girhard *et al.*, 2010)  CYP109D1 (Khatri *et al.*, 2010)  CYP109E1 (Jóźwik *et al.*, 2016)(Putkaradze *et al.*, 2017) |

Thankfully, the annotation was done by Professor D. Nelson (Health Science Center, University of Tennessee) based on the P450 nomenclature.

Table S2. List of substrates used in this study and overview of the results obtained with CYP109Q5.

|  | **Substrate** | **CYP109Q5** |
| --- | --- | --- |
| **Norisoprenoids, Mono- and Sesquiterpenes** | |  |
|  | β-Ionone | +++ |
|  | Vinylionol | ++ |
|  | α-Humulene | + |
|  | β-Bisabolene | + |
|  | Premnaspirodiene | + |
|  | Longifolene | + |
|  | Valencene | + |
|  | Nootkatone | + |
|  | Caryophyllenoxide | + |
|  | β-Caryophyllene | ++ |
|  | Aromadendrene | Traces |
|  | Zerumbone | ++ |
|  | β-Damascone | +++ |
|  | α-Ionone | +++ |
|  | Pseudoionone | ++ |
|  | α-Terpineol | + |
|  | Carvone | + |
|  | Linalool | + |
|  | Terpinolene | Traces |
|  | Geranylacetone | +++ |
|  | Nerolidol | + |
|  | Camphor | + |
|  | Pinene | - |
|  | Limonene | + |
|  | Thujene | + |
|  | Nerol | + |
| **NSAIDs (*nonsteroidal anti-inflammatory drugs*)** | |  |
|  | Diclofenac | ++ (s) |
|  | Naproxen | - |
|  | Ibuprofen | ++ (s) |
|  | Mefenamic acid | ++ (s) |
| **Aromatics** | |  |
|  | Fluorene | - |
|  | Eugenol | - |
|  | Guajacol | - |
|  | *cis*-Stilbene | + |
|  | Phenanthrene | - |
|  | *trans*-Stilbene | - |
|  | Cumol | - |
| **Fatty acids** | |  |
|  | Lauric acid (C12) | Traces |
|  | Myristic acid (C14) | Traces |
|  | Palmitic acid (C16) | Traces |
| **Steroids** | |  |
|  | Progesterone | ++ |
|  | Testosterone | ++ |
| **Further substrates** | |  |
|  | 7-Ethoxycoumarine | - |
|  | 3-Chloro-4-methoxyaniline | - |
|  | Cinnamic acid | - |
|  | Phenylacetic acid | - |
|  |  |  |

The substrates were each tested with the functional active redox partner CamA and CamB. If no product standard was available, the conversions were estimated on the basis of the ratios of the product peak areas to the internal standard compared to the residual substrate. - (no conversion), + (< 10%), ++ (10 - 50%), +++ (> 50%), s (selective, 1 product).

Table S3. Data Collection and Refinement Statistics for CYP109Q5.

|  | CYP109Q5 |
| --- | --- |
| Beamline | Diamond I04 |
| Wavelength (Å) | 0.97950 |
| Resolution (Å) | 55.02-1.55 (1.58-1.55) |
| Space Group | *P*2_1_1 |
| Unit cell (Å) | a = 44.69; b = 55.02; c = 74.80  α = γ = 90.00°; β = 91.67 |
| No. of molecules in the asymmetric unit | 1 |
| Unique reflections | 52220 (2586) |
| Completeness (%) | 98.9 (99.8) |
| *R*_merge_ (%) | 0.05 (0.53) |
| *R*_p.i.m._ | 0.04 (0.45) |
| Multiplicity | 3.9 (4.0) |
| <*I*/*σ(I)>* | 11.6 (2.0) |
| Overall *B* factor from Wilson plot (Å^2^) | 19 |
| CC_1/2_ | 1.00 (0.82) |
| *R_cryst_/ R_free_* (%) | 18.5/22.0 |
| r.m.s.d 1-2 bonds (Å) | 0.012 |
| r.m.s.d 1-3 angles (^o^) | 1.59 |
| Avge main chain B (Å^2^) | 25 |
| Avge side chain B (Å^2^) | 27 |
| Avge water B (Å^2^) | 34 |

Numbers in brackets refer to data for highest resolution shells.

Table S4. Results of the rational enzyme design of CYP109Q5.

| **Position** | **Analysis** | **Exchange** | | **Effect** | | |
| --- | --- | --- | --- | --- | --- | --- |
| **Single variants** |  |  |  | |  |  |
| **Q35** | Anchor for fatty acids? | **V** | | **↗** | |  |
| **V80** | *hotspot*-Position 57^3DM^ | **G, A, I, L** | | **↑↑ (G, A)**  **→ (L)**  **↓ (I)** | |  |
| **T229** | *hotspot*-Position 211^3DM^ | **S, L** | | **↑** (especially with norisoprenoids) | |  |
| **I232** | Terpene Hydroxylases with 100% Leu | **L** | | **→** | |  |
| **A233** | *hotspot-*Position and contact | **G, V** | | **→** | |  |
| **T237** | conserved: O_2_ activation? | **S** | | **↑** | |  |
| **A280** | Five residues after ExxR motif | **V, L, I, F** | | **↑↑ (I, V)**  **↓ (L, F)** | |  |
| **I283** | Contact with substrates | **L, V** | | **↑↑** | |  |
| **F381** | *hotspot*-Position for regioselectivity | **V** | | **↗** | |  |
| **Double variants** | **Combination** |  | |  | |  |
| **V80A** | **A280L, A280I, A280V** |  | | **↑↑** | |  |
| **V80G** | **A280I, A280V** |  | | **↑↑** | |  |
| **V80G** | **I283L, T237S ^a)^** |  | | **↑** | |  |
| **V80G** | **T229S, T229L ^b)^** |  | | **-** | |  |
| **A280V** | **T229L** |  | | **↑↑** | |  |

The variants were first tested *in vitro* with cell lysate (2 μM P450) and CamA and CamB as redox partners for 24 h with the terpenes β-ionone, vinylionol, valencene, β-bisabolene, premnaspirodiene, longifolene and α-humulene. Addressed positions are presented with the respective exchange carried out and with the results from the bioinformatic analysis. Based on the biotransformations the following effects were observed: ↓ negative effect; → wild type like; ↗ low positive effect; ↑ positive effect; ↑↑ strong positive effect.

a) low functional expression

b) no functional P450 after expression

| **Construct** | **Change in Absorption** | | | **Dilution** | **P450 concentration [µM]** |
| --- | --- | --- | --- | --- | --- |
|  | 420 nm | 450 nm | 490 nm |  |  |
| WT | 0.034 | 0.115 | 0.006 | 20 | **24.0** |
| V80G | 0.207 | 0.04 | 0.019 | 20 | **4.6** |
| A280I | 0.039 | 0.176 | 0.009 | 20 | **36.7** |
| A280V | 0.03 | 0.147 | -0,019 | 20 | **36.5** |
| I283L | 0.045 | 0.058 | 0 | 20 | **12.7** |
| V80A/A280I | 0.176 | 0.072 | -0.001 | 20 | **16.0** |
| V80A/A280L | 0.007 | 0.052 | -0.007 | 20 | **13.0** |
| V80G/A280I | 0.213 | 0.068 | 0.009 | 20 | **13.0** |
| A280V/T229L | 0.011 | 0.092 | -0.003 | 20 | **20.9** |

Table S5. CO difference spectra of the IEX purified CYP109Q5 variants.

Table S6. Biocatalysts with activity towards the investigated norisoprenoid β-ionone.

| **Microbial biocatalyst** | **Product(s)** | **Reference** |
| --- | --- | --- |
| P450 BM3 variants, CYP102A7, CYP109B1, CYP109D1, CYP267B1, *Streptomyces* strains | 4-Hydroxy-β-ionone | (Lutz-Wahl *et al.*, 1998; Urlacher *et al.*, 2006; Dietrich *et al.*, 2008; Girhard *et al.*, 2010; Khatri *et al.*, 2011; Zehentgruber *et al.*, 2012; Zhang *et al.*, 2015; Litzenburger and Bernhardt, 2016) |
| P450 Soy C, SU1, SU2 | 4-Hydroxy-β-ionone & n.d. | (Celik *et al.*, 2005) |
| CYP260A1 | 4-Hydroxy-β-ionone & epoxides | (Litzenburger and Bernhardt, 2016) |
| *Aspergillus niger* strains | 2- and 4-Hydroxy-β-ionone & overoxidation products | (Mikami *et al.*, 1978; Sode *et al.*, 1989; Larroche *et al.*, 1995; Grivel and Larroche, 2001) |
| CYP101B1, CYP101C1 | 2- and 3-Hydroxy-β-ionone | (Bell and Wong, 2007; Bell *et al.*, 2010; Ma *et al.*, 2011; Hall and Bell, 2015) |
| CYP264B1 | 3-Hydroxy-β-ionone | (Ly *et al.*, 2012) |
| CYP109Q5 | 2-, 3- and 4-Hydroxy-β-ionone | This study |

n.d.: not determined.

Table S7. Primers used in this study.

| **Primer** | **Sequence (5’ – 3’) Primer forward/ Primer reverse** |
| --- | --- |
| C.apic_CYP109Q5  (*Nde*I + *Hind*III) | GACTACTA**CATATG**ATCGCCGACATCGACATCACC/  CTATGTAAGCTTTAGGCGCTCGCCACCGTGGCCCG (more common stop-codon inserted) |
| **Gibson Assembly**^®^ **Primer** |  |
| GA_CamB_pBAD18 | GGCTAGCAGGAGGATTAACCATGTCTAAAGTAGTGTATGTGTCACATGATGG/ TCGACTCTAGACCCGGGTTATTACCATTGCCTATCGGGAACATCG |
| GA_V_pBAD18_Cam | CTGAACTGAGTAGTGCCTGATAATCTAGACTGCAGAAGCTTGGC/  ACATACACTACTTTAGACATGGTTAATCCTCCTGCTAGCC |
| GA_Pro2_Cam | TTCCCGATAGGCAATGGTAATAACCCGGGTCTAGAGTCGAC/  ACGTTGTCGTTTGCGTTCATATGTAATCCTCCTGGTACCAAAAAAGAG |
| GA_CamA_pBAD18 | TGGTACCAGGAGGATTACATATGAACGCAAACGACAACGT/  AGCTTCTGCAGTCTAGATTATCAGGCACTACTCAGTTCAGCTTTG |
| **QuikChange® Primer** |  |
| CYP109Q5_Q35V | GGCCGCCTGCAGCCC**GTG**GGCTTCATGGCCGTGGGG/  CCCCACGGCCATGAAGCC**CAC**GGGCTGCAGGCGGCC |
| CYP109Q5_V80G | CATGCCTCCCTCCATC**GGT**CAGGTCGATCCGCCCCGC/  GCGGGGCGGATCGACCTG**ACC**GATGGAGGGAGGCATG |
| CYP109Q5_V80A | CATGCCTCCCTCCATC**GCC**CAGGTCGATCCGCCCCGC/  GCGGGGCGGATCGACCTG**GGC**GATGGAGGGAGGCATG |
| CYP109Q5_V80I | CATGCCTCCCTCCATC**ATT**CAGGTCGATCCGCCCCGC/  GCGGGGCGGATCGACCTG**AAT**GATGGAGGGAGGCATG |
| CYP109Q5_V80L | CATGCCTCCCTCCATC**CTG**CAGGTCGATCCGCCCCGC/  GCGGGGCGGATCGACCTG**CAG**GATGGAGGGAGGCATG |
| CYP109Q5_T229S | CTGAGCTTCGCGAAC**TCG**CTGCTCATCGCCGGCAAC/  GTTGCCGGCGATGAGCAG**CGA**GTTCGCGAAGCTCAG |
| CYP109Q5_T229L | CTGAGCTTCGCGAAC**CTG**CTGCTCATCGCCGGCAAC/  GTTGCCGGCGATGAGCAG**CAG**GTTCGCGAAGCTCAG |
| CYP109Q5_T229D | CTGAGCTTCGCGAAC**GAT**CTGCTCATCGCCGGCAAC/  GTTGCCGGCGATGAGCAG**ATC**GTTCGCGAAGCTCAG |
| CYP109Q5_I232L | GCGAACACGCTGCTC**CTG**GCCGGCAACGAGACGACG/  CGTCGTCTCGTTGCCGGC**CAG**GAGCAGCGTGTTCGC |
| CYP109Q5_A233G | GAACACGCTGCTCATC**GGC**GGCAACGAGACGACGACG/  CGTCGTCGTCTCGTTGCC**GCC**GATGAGCAGCGTGTTC |
| CYP109Q5_A233V | GAACACGCTGCTCATC**GTG**GGCAACGAGACGACGACG/  CGTCGTCGTCTCGTTGCC**CAC**GATGAGCAGCGTGTTC |
| CYP109Q5_T237S | CATCGCCGGCAACGAG**TCG**ACGACGAGCCTCATCGGC/  GCCGATGAGGCTCGTCGT**CGA**CTCGTTGCCGGCGATG |
| CYP109Q5_A280V | CGCTACGAGAGCCCG**GTG**CAGTGCATCTTCCGGCAG/  CTGCCGGAAGATGCACTG**CAC**CGGGCTCTCGTAGCG |
| CYP109Q5_A280L | CGCTACGAGAGCCCG**CTG**CAGTGCATCTTCCGGCAG/  CTGCCGGAAGATGCACTG**CAG**CGGGCTCTCGTAGCG |
| CYP109Q5_A280I | CGCTACGAGAGCCCG**ATT**CAGTGCATCTTCCGGCAG/  CTGCCGGAAGATGCACTG**AAT**CGGGCTCTCGTAGCG |
| CYP109Q5_A280F | CGCTACGAGAGCCCG**TTT**CAGTGCATCTTCCGGCAG/  CTGCCGGAAGATGCACTG**AAA**CGGGCTCTCGTAGCG |
| CYP109Q5_I283L | GAGCCCGGCGCAGTGC**CTG**TTCCGGCAGACCATGACG/  CGTCATGGTCTGCCGGAA**CAG**GCACTGCGCCGGGCTC |
| CYP109Q5_I283V | GAGCCCGGCGCAGTGC**GTG**TTCCGGCAGACCATGACG/  CGTCATGGTCTGCCGGAA**CAC**GCACTGCGCCGGGCTC |
| CYP109Q5_F381A | GTGGTCGCCGTCGTTC**GCG**ATCCGGTCGCCGAGCACG/  CGTGCTCGGCGACCGGAT**CGC**GAACGACGGCGACCAC |

Table S8. Reaction batches for the amplification from *Chondromyces apiculatus* genomic DNA.

| **Component** | **Volume** | **Final concentration** |
| --- | --- | --- |
| 10x KOD HS buffer | 5 µL | 1x |
| Primer forward and reverse | each 1.5 µL | each 0.3 µM |
| 10 mM dNTP-Mix | 2.5 µL | each 500 µM |
| 25 mM MgSO_4_ | 4 µL | 2 mM |
| DMSO | 2.5 µL | 5% v/v |
| Genomic DNA | 20 µL | ~ 3 – 4 ng µL^-1^ |
| KOD HS Polymerase | 1 µL | 1 U µL^-1^ |
| ddH_2_O | to 50 µL | - |

| **Step** | **Temperature** | **Duration** |
| --- | --- | --- |
| 1. Initial denaturation | 95°C | 2 min |
| 2. Denaturation | 95°C | 30 s |
| 3. Annealing | 55°C | 15 s |
| 4. Elongation | 70°C | 1 min 30 s |
| Further cycles from step 2. (30x) |  |  |
| 5. Final elongation | 70°C | 5 min |
| 6. Hold | 8°C | ∞ |

Table S9. Expression conditions for the applied redox proteins within this study.

| **Construct** | ***E. coli* expression strain** | **Expression temperature** | **Inductor** |
| --- | --- | --- | --- |
| pET-28a(+)_**CamB** | BL21(DE3) | 25°C | 0.3 mM IPTG, 0.3 mM FeSO_4_ |
| pkkHC_**AdX** | JM109 | 20°C | 0.3 mM IPTG |
| pET-28a(+)_**CamA** | BL21(DE3) | 25°C |  |
| pET-16b_**FdR** | BL21(DE3) | 20°C | 0.2 mM IPTG |
| pET-11a_**FdX** | BL21(DE3) | 20°C |  |

Table S10. QuikChange® PCR approaches.

| **Component** | **Volume** | **Final concentration** |
| --- | --- | --- |
| 10x KOD HS buffer | 5 µL | 1x |
| Primer forward and reverse | each 1 µL | each 0.2 µM |
| 2 mM dNTP-Mix | 5 µL | each 200 µM |
| 25 mM MgSO_4_ | 4.5 µL | 2.25 mM |
| DMSO | A) 2.5 µL  B) 5 µL | A) 5% v/v  B) 10% v/v |
| Template DNA | 0.5 µL | ~ 1 ng µL^-1^ |
| KOD HS Polymerase | 1 µL | 1 U µL^-1^ |
| ddH_2_O | to 50 µL | - |

A) Approach 1: Position Q35, T229, I232, A233, T237, A280

B) Approach 2: Position V80, I283, F381

| **Step** | **Temperature** | **Duration** |
| --- | --- | --- |
| 1. Initial denaturation | 95°C | 2 min |
| 2. Denaturation | 95°C | 30 s |
| 3. Annealing | A) 60°C  B) 66°C | 10 s |
| 4. Elongation | 70°C | 3 min |
| Further cycles from step 2. (20x) |  |  |
| 5. Final elongation | 70°C | 5 min |
| 6. Hold | 8°C | ∞ |

Table S11. Gibson Assembly® approach.

| **Component** | **Volume** | **Final concentration** |
| --- | --- | --- |
| 10x KOD HS buffer | 5 µL | 1x |
| Primer forward and reverse | each 1.5 µL | each 0.3 µM |
| 10 mM dNTP-Mix | 2.5 µL | each 400 µM |
| 25 mM MgSO_4_ | 4 µL | 2 mM |
| DMSO | 2.5 µL | 5% v/v |
| Template DNA | 0.75 µL | ~ 1 – 2 ng µL^-1^ |
| KOD HS Polymerase | 1 µL | 1 U µL^-1^ |
| ddH_2_O | to 50 µL | - |

| **Step** | **Temperature** | **Duration** |
| --- | --- | --- |
| 1. Initial denaturation | 95°C | 2 min |
| 2. Denaturation | 95°C | 30 s |
| 3. Annealing | 5°C under T_m_ Primer | 30 s |
| 4. Elongation | 72°C | 30 s per kb |
| Further cycles from step 2. (34x) |  |  |
| 5. Final elongation | 72°C | 4 min |
| 6. Hold | 8°C | ∞ |

Gibson Assembly® Mix

| **Component** | **Volume** |
| --- | --- |
| 5x Iso reaction buffer (500 mM Tris/HCl pH 7.5, 25% PEG-8000, 50 mM MgCl, 50 mM DTT, 5 mM NAD, each 1 mM dATP, dCTP, DGTP, dTTP) | 320 µL |
| T5 Exonuclease (10 U µL^-1^) | 0.64 µL |
| *Phusion*®High-Fidelity DNA-Polymerase (2 U µL^-1^) | 20 µL |
| *Taq* DNA Ligase (40 U µL^-1^) | 160 µL |
| ddH_2_0 | to 1.2 mL |
| Aliquoted to 15 µL, stored at -20°C |  |

Table S12. GC programs used in this study.

| **Substrate** | **Column** | **Deriva-tization** | **Injection** | **Temperature program** |
| --- | --- | --- | --- | --- |
| β-Ionone, Vinylionol, Pseudoionone, α-Ionone, β-damascone, α-Humulene, β-Bisabolene, Premnaspirodiene, Longifolene, Valencene, Caryophylleneoxide, β-Caryophyllene, Aromadendrene, Zerumbone, Nootkatone, Fluorene, Phenanthrene, 7‑Ethoxycoumarin | HP-1ms-ui | No | *In vitro* (2 µL, Split 1.5) | 130°C, 1 min hold  10°C min^-1^ to 185°C, 2 min hold  10°C min^-1^ to 220°C  50°C min^-1^ to 320°C, 3 min hold |
| Nerolidol, Camphor, Pinene, Limonene, α-Terpineol, Carvone, Linalool, Terpinolene, Geranylacetone, Thujene, Nerol, *cis*- and *trans*-Stilbene | HP-1ms-ui | No | *In vitro* (2 µL, Split 1.5) | 85°C, 1 min hold  10°C min^-1^ to 120°C  7°C min^-1^ to 170°C  15°C min^-1^ to 200°C  50°C min^-1^ to 320°C, 3 min hold |
| Eugenol, 3-chloro-4-methoxyanilin | HP-1ms-ui | No | *In vitro* (2 µL, Split 1.5) | 100°C, 1 min hold  10°C min^-1^ to 200°C, 1 min hold  50°C min^-1^ to 320°C, 3 min hold |
| Guajacole | HP-1ms-ui | No | *In vitro* (2 µL, Split 1.5) | 55°C, 2 min hold  10°C min^-1^ to 150°C, 1 min hold  50°C min^-1^ to 320°C, 3 min hold |
| Cumol | HP-1ms-ui | No | *In vitro* (2 µL, Split 1.5) | 55°C, 2 min hold  8°C min^-1^ to 120°C,  12°C min^-1^ to 170°C,  50°C min^-1^ to 320°C, 3 min hold |
| Lauric acid, Myristic acid | DB-5 | Yes | *In vitro* (1 µL, Split 5) | 130°C, 1 min hold  10°C min^-1^ to 250°C, 1 min hold  65°C min^-1^ to 320°C, 3 min hold |
| Palmitic acid, Naproxen, Mefenamic acid | DB-5 | Yes | *In vitro* (1 µL, Split 5) | 150°C, 1 min hold  10°C min^-1^ to 280°C, 1 min hold  65°C min^-1^ to 320°C, 3 min hold |
| Cinnamic acid, Phenylacetic acid, Ibuprofen | DB-5 | Yes | *In vitro* (1 µL, Split 5) | 90°C, 1 min hold  12°C min^-1^ to 280°C, 1 min hold  65°C min^-1^ to 320°C, 3 min hold |

Fig. S1. IEX purification of CYP109Q5. *Magenta*: injection time; *Blue*: total protein (280 nm); *Red*: P450 (420 nm) and fractionation; *Green*: concentration of elution buffer with 1 M NaCl (7, 30, 100% steps); *Cyan*: conductivity measurement.


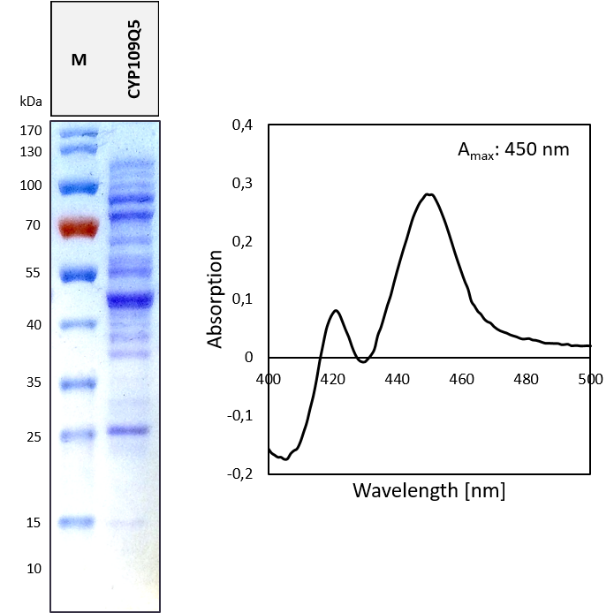


Fig. S2. SDS-PAGE und CO difference spectra of the purified CYP109Q5 wild type (44 kDa). CYP109Q5 was partially purified through IEX for biotransformation studies. Purity of the P450 was estimated to be greater than 60% by SDS–PAGE analysis.


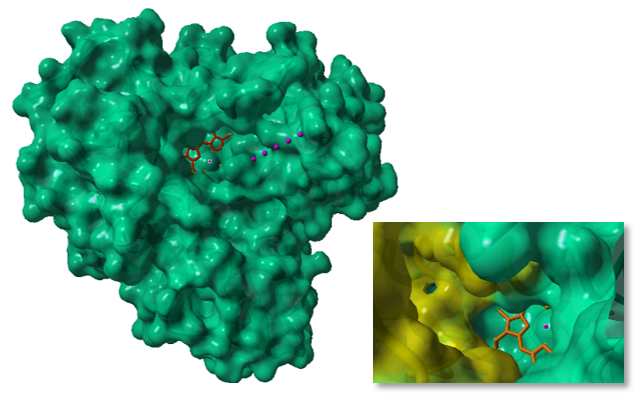


Fig. S3. Crystal structure of CYP109Q5. The surface display is shown to indicate the substrate entrance with the heme in *orange*. The active site contains a big cavity (surface display, *yellow*) which presumably allow binding of large linear substrates to the enzyme.


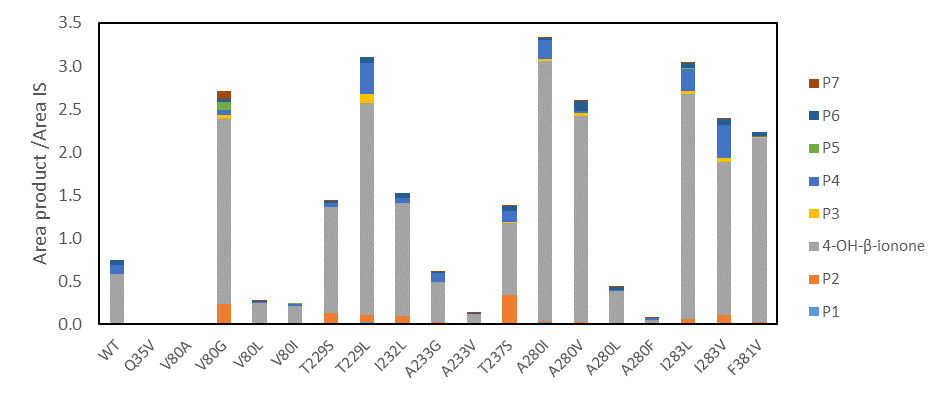


**1**

*

*


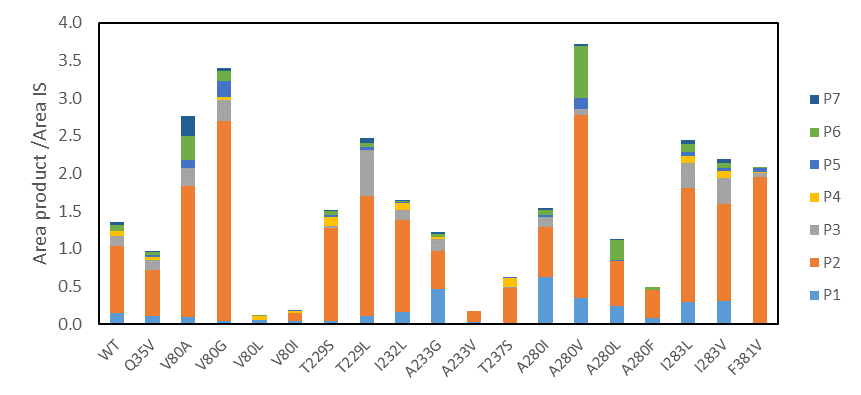


**2**


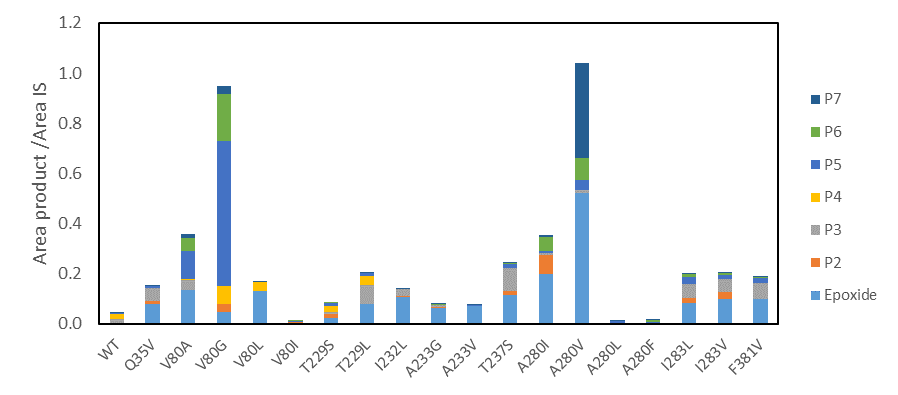


**3**


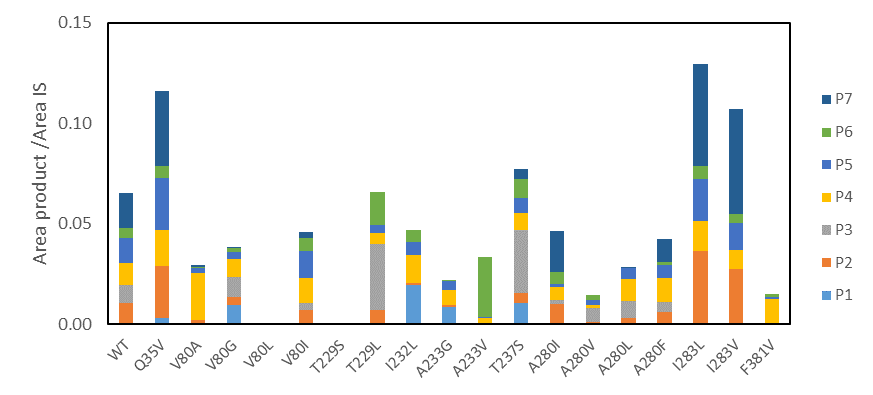


**4**

*

*


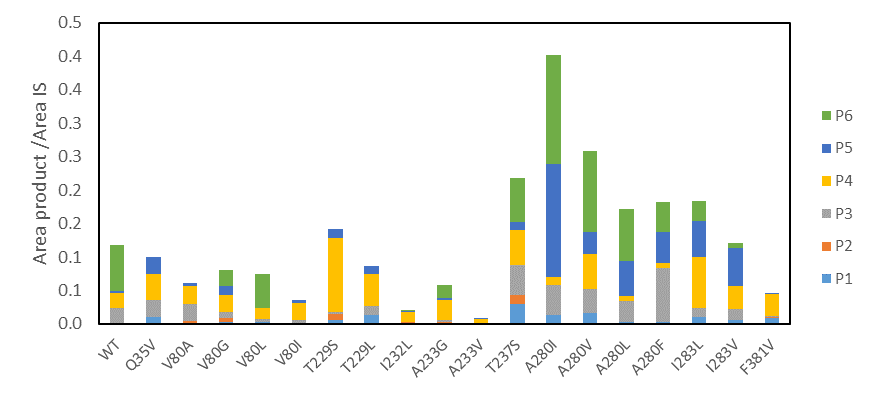


**5**


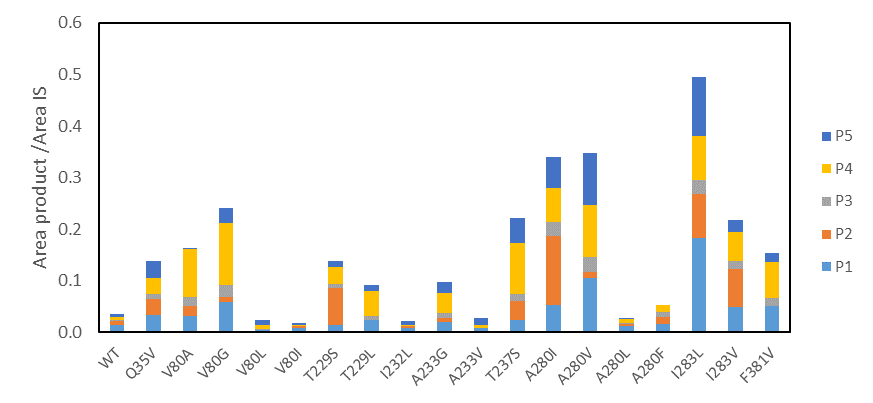


**6**


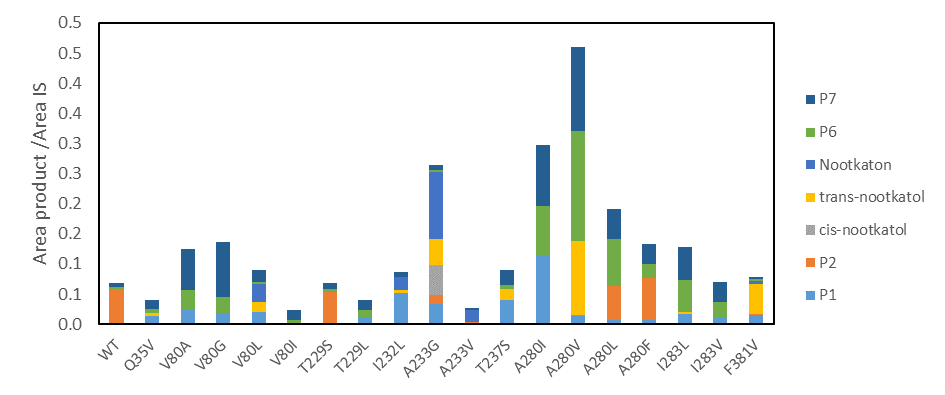


**7**

Fig. S4. Initial screening of the CYP109Q5 single variants (lysate) with different terpenes. The reactions were carried out with 2 μM lysate and the redox partner system CamA/B for 24 h. Formed products were analyzed by GC-FID and GC-MS. 0.2 mM Carvone was used as internal standard (IS). Products were numbered consecutively according to the order based on retention time for each substrate. In case of possible identification *via* product standards trivial names are given. 1: β-Ionone; 2: Vinylionol; 3: α-Humulene; 4: Bisabolene; 5: Premnaspirodiene; 6: Longifolene; 7: Valencene.


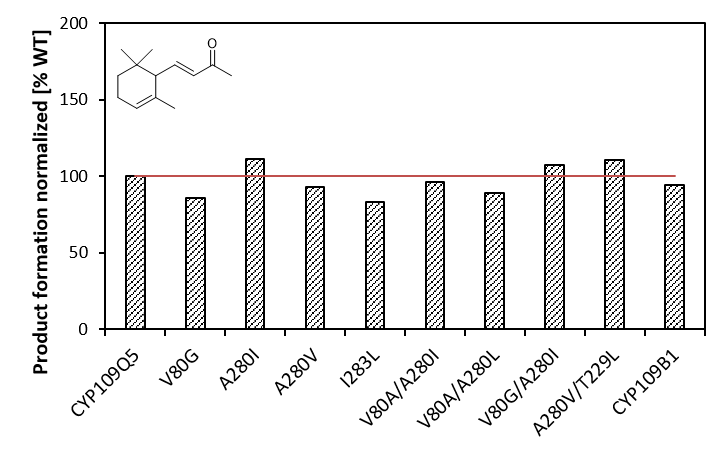

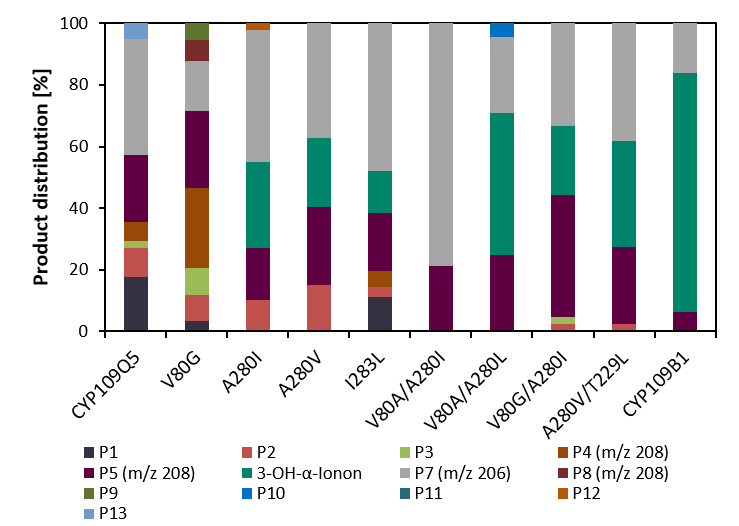

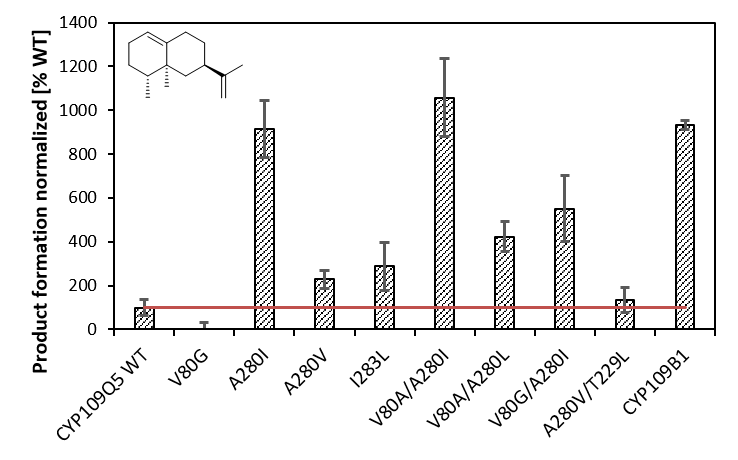

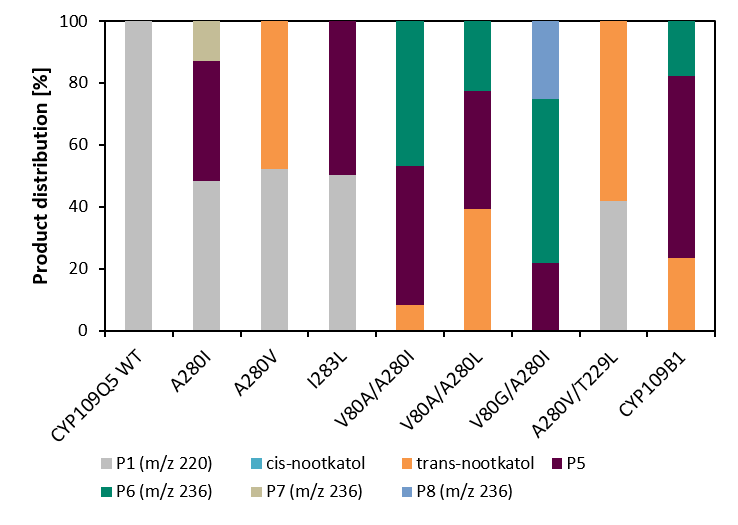

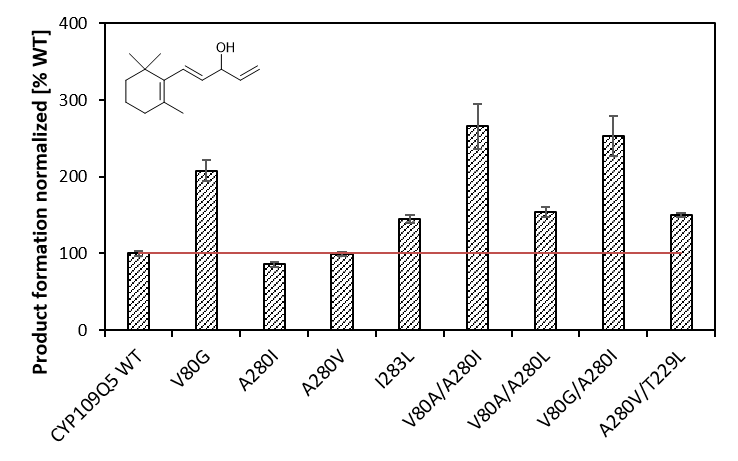

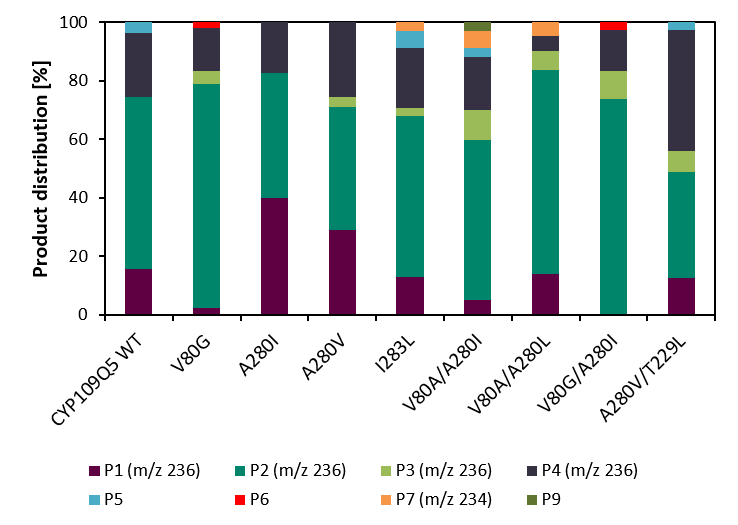

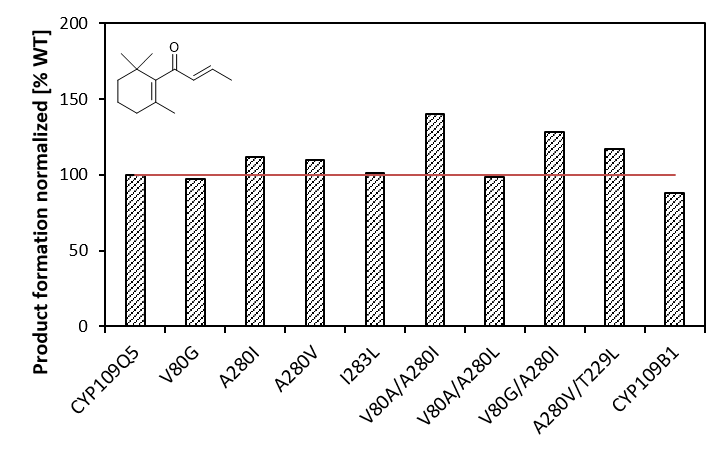

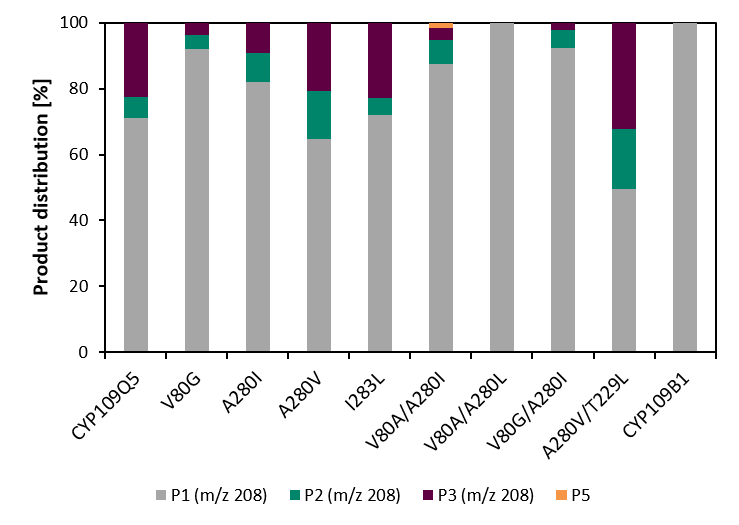


A)

B)

C)

D)


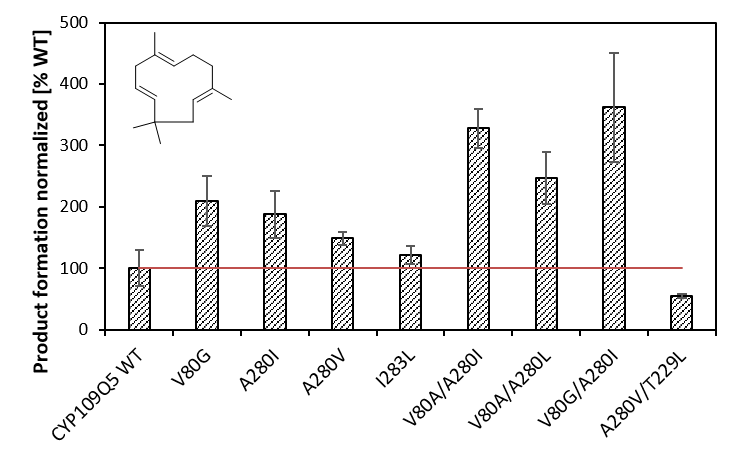

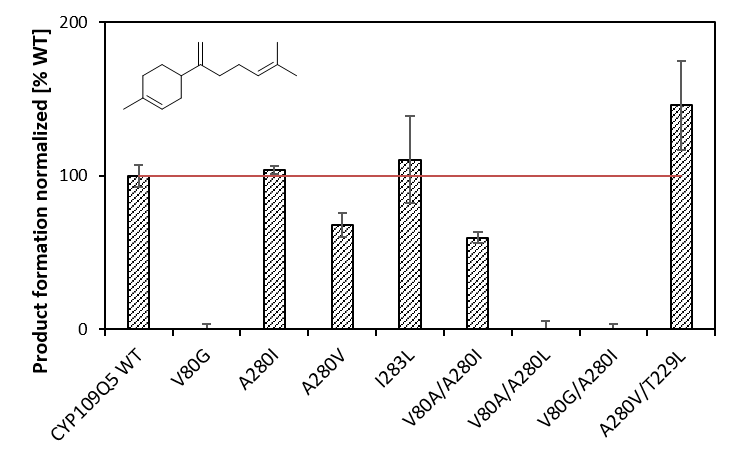

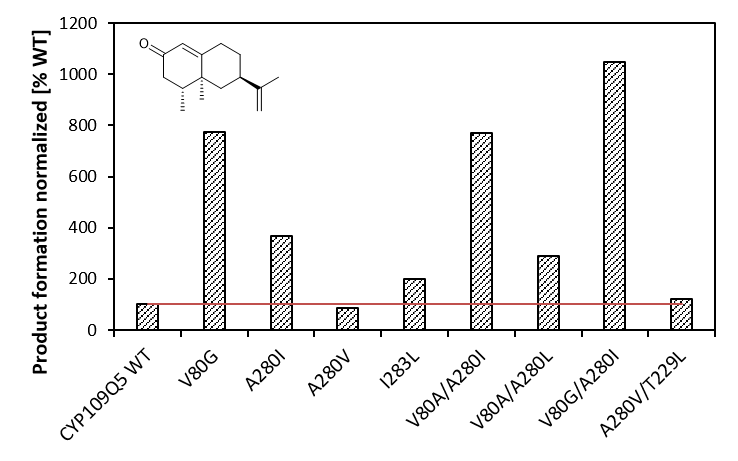

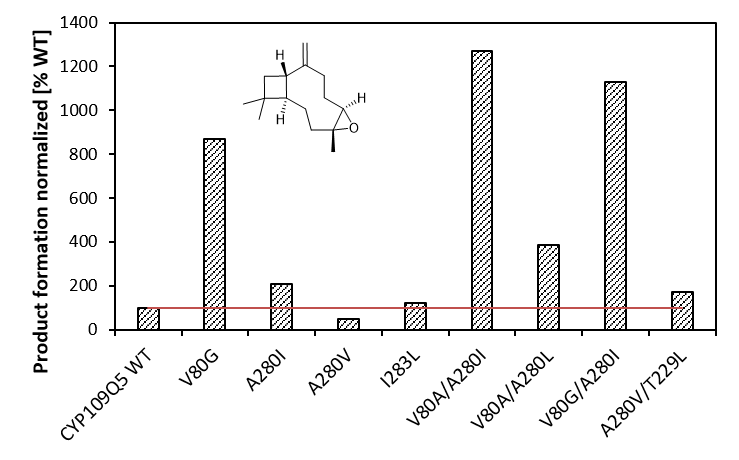

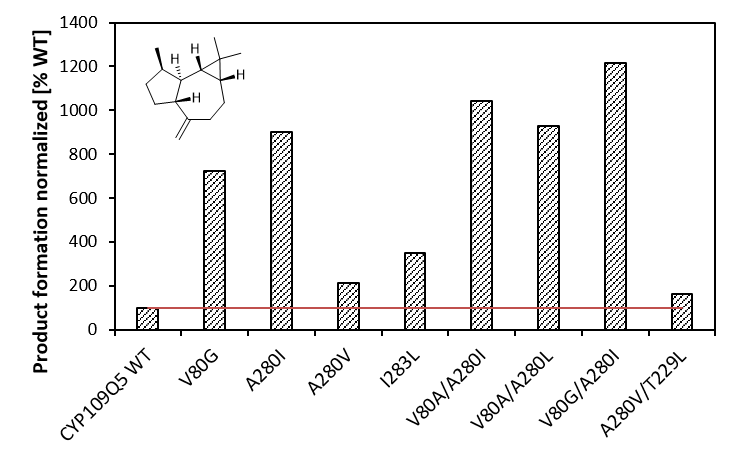


E)

F)

G)

H)


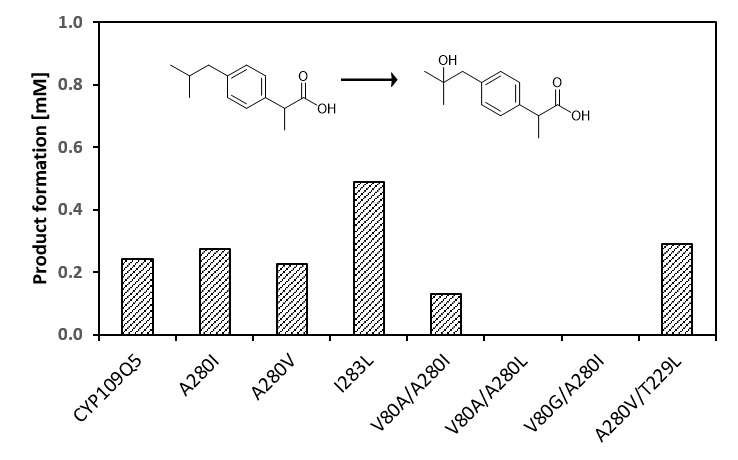


I)

J)

L)

K)


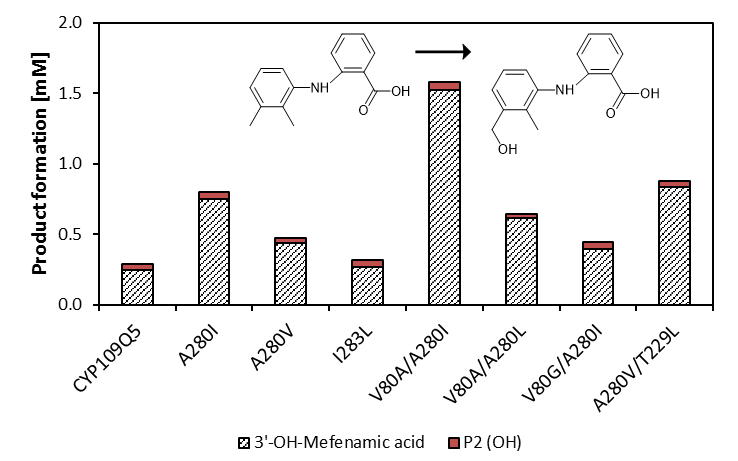

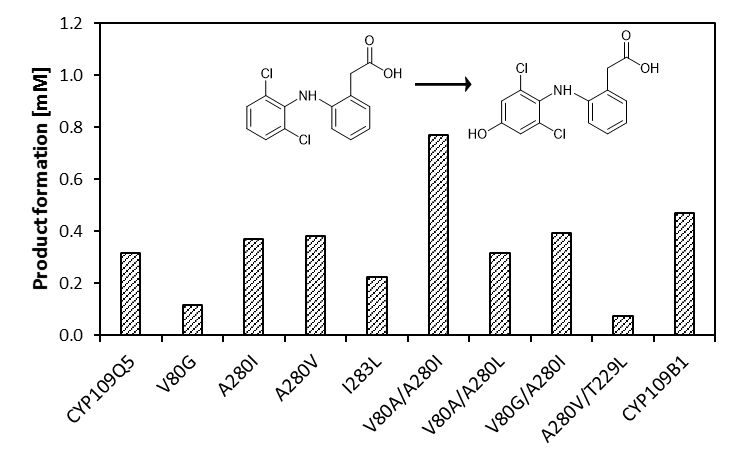


Fig. S5. *In vitro* screening of the purified CYP109Q5 variants and/or CYP109B1 for 2 h. A: Valencene; B: Vinylionol; C: β-Damascone; D: α-ionone; E: α-humulene; F: β-Bisabolene; G: Nootkatone; H: Caryophyllenoxide; I: Aromadendrene; J: Diclofenac; K: Mefenamic acid; L: Ibuprofen. The mass m/z is given for clearly assignable products. Measurements were performed in triplicates except of C), D), G) to L). Products were numbered consecutively according to the order based on retention time for each substrate. In case of possible identification *via* product standards trivial names are given. Following values of area product divided by area internal standard were reached with the purified wild-type enzyme: A: 0.08; B: 2.07; C: 3.46; D: 3.27; E: 0.46; F: 0.35; G: 0.47; H: 0.31; I: 0.08.

Fig. S6. Investigation of L-arabinose concentration and temperature for the expression of the dual-vector system CYP109Q5 CamA/B. The diagram shows the *in vivo* activities of different expressions with CYP109Q5 resting cells 0.1 g_cww_ mL^-1^, with 30 mM glucose and with 5 mM β-ionone (250 mM stock solution in DMSO) for 24 h at 30°C and 180 rpm.

Indol

Fig. S7. Semi-preparative HPLC purification of the CYP109Q5 A280V/T229L preparative biotransformation with β-ionone.


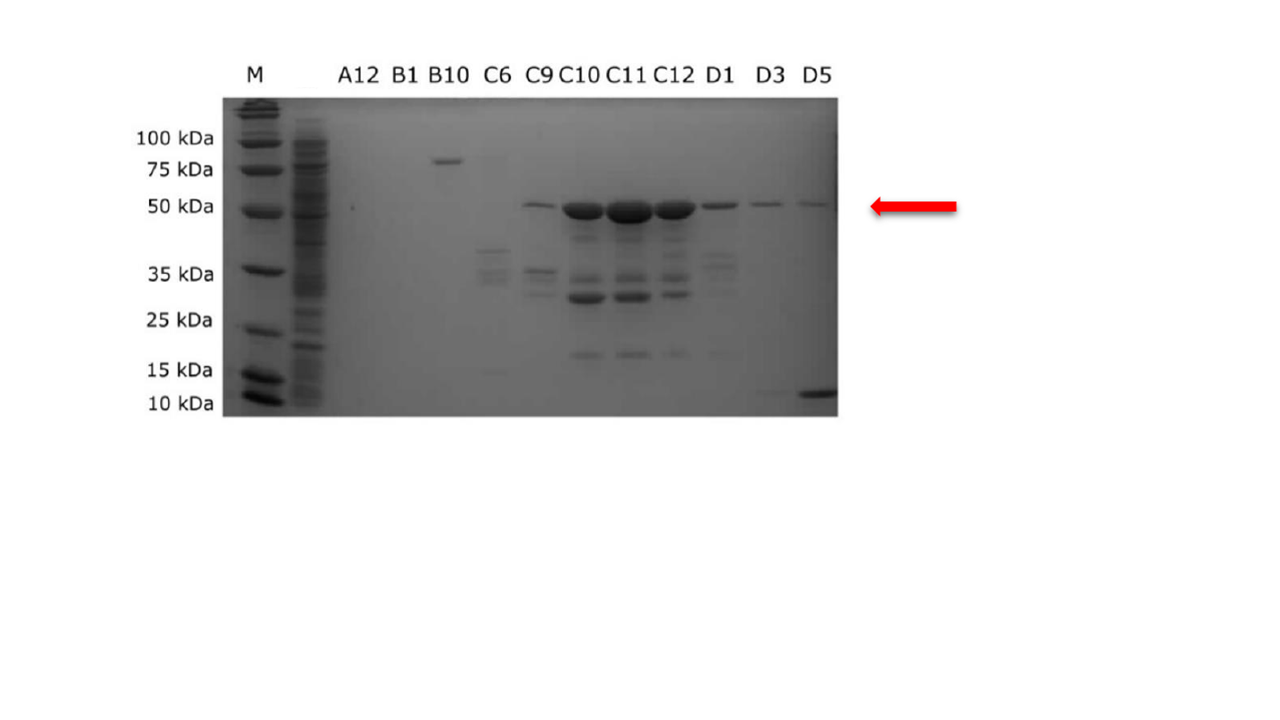


**Fig. S8**. SDS-PAGE analysis of size exclusion chromatography of CYP109Q5 expressed using the codon-optimized gene in the pET-28a(+) vector. **M:** low molecular weight markers**;** Lanes **A12** – **D5** correspond to sequentially eluted fractions. The protein of interest is indicated by the red arrow. Fractions C12 – D3 were pooled and concentrated for use in crystallization screens.


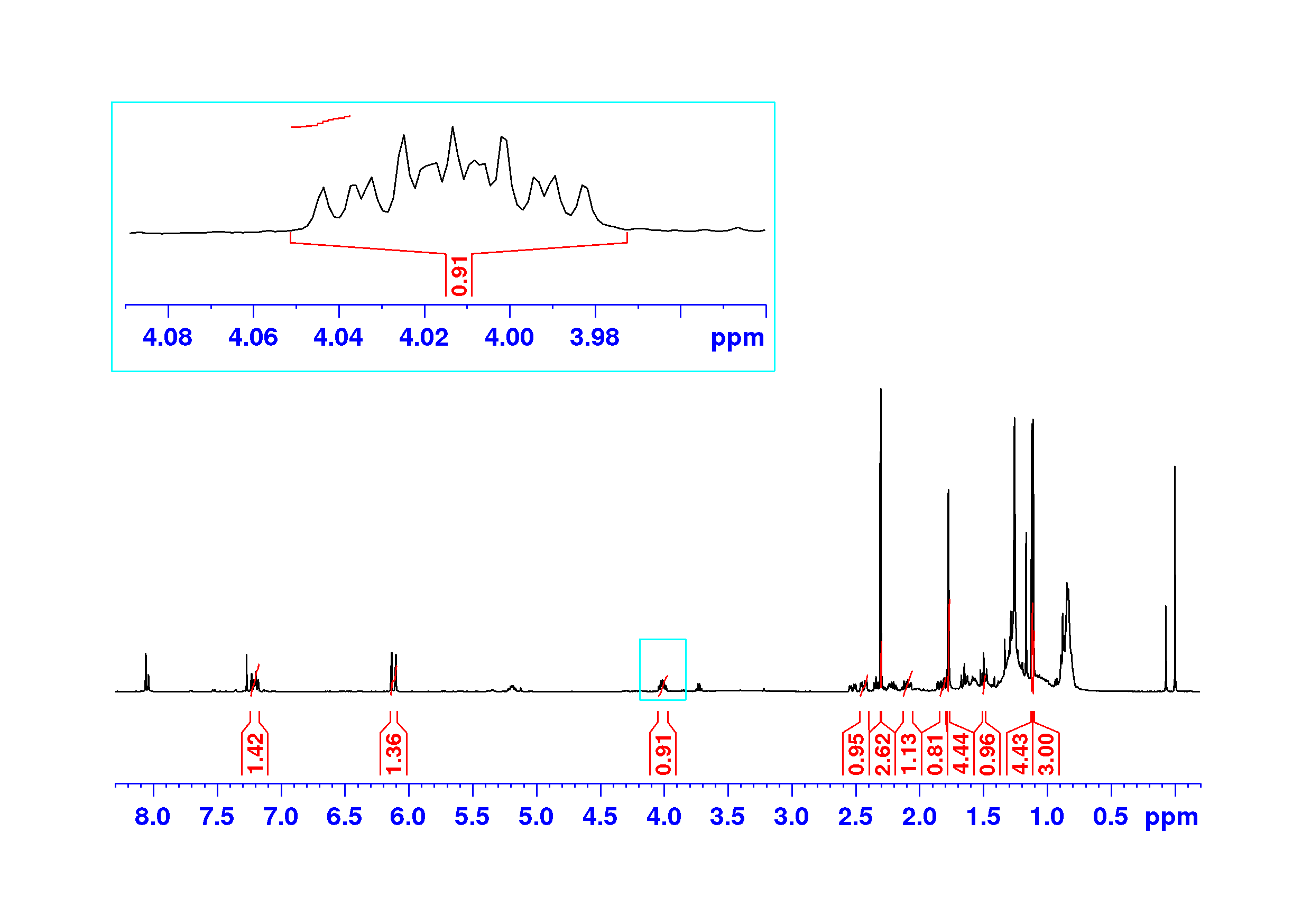


FA

CDCl_3_

TMS


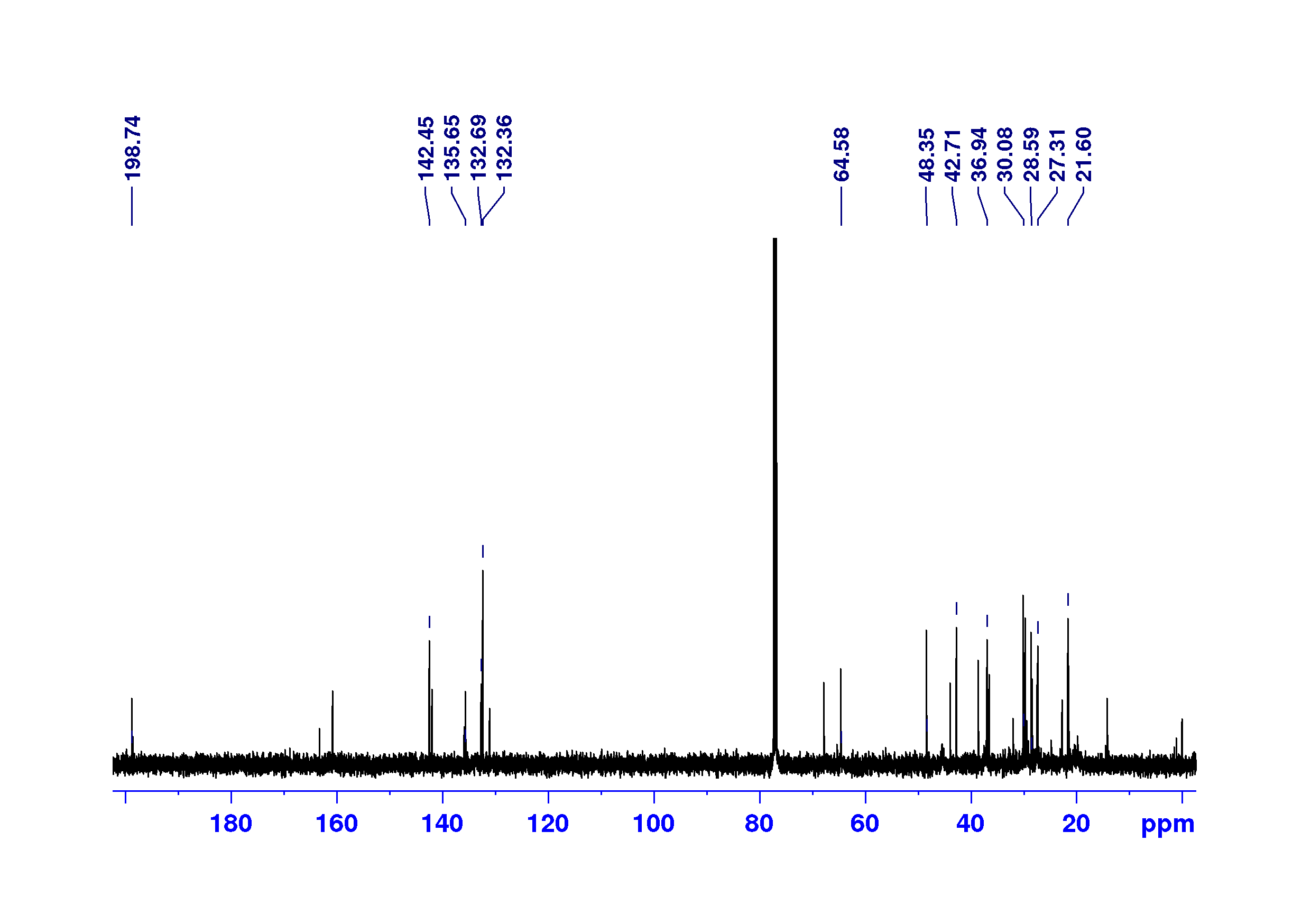


CDCl_3_

TMS

FA

Fig. S9. ^1^H and ^13^C-NMR spectra of 3-hydroxy-β-ionone. FA: Formic acid.


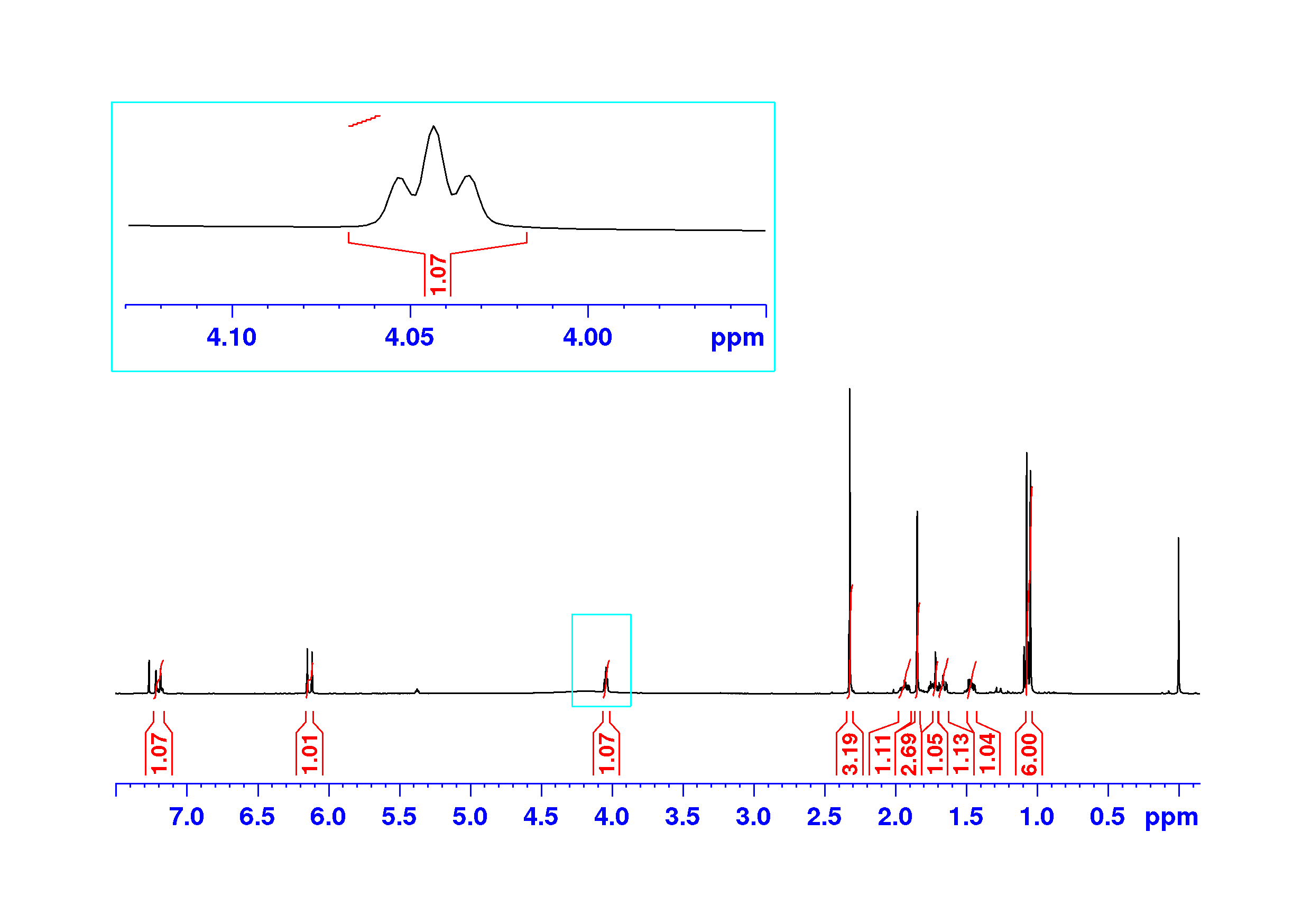


CDCl_3_

TMS


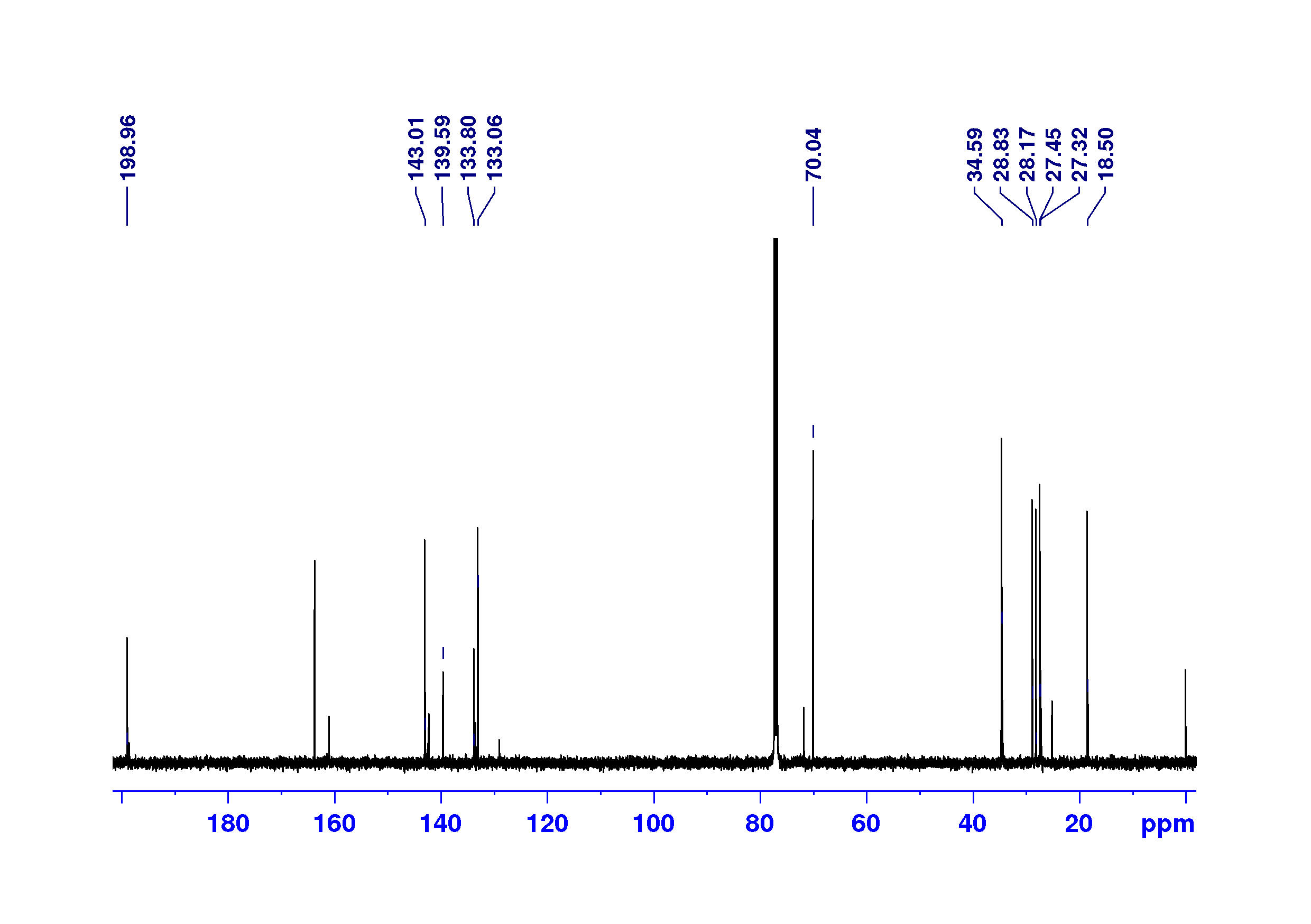


CDCl_3_

TMS

FA

Fig. S10. ^1^H and ^13^C-NMR spectra of 4-hydroxy-β-ionone. FA: Formic acid.


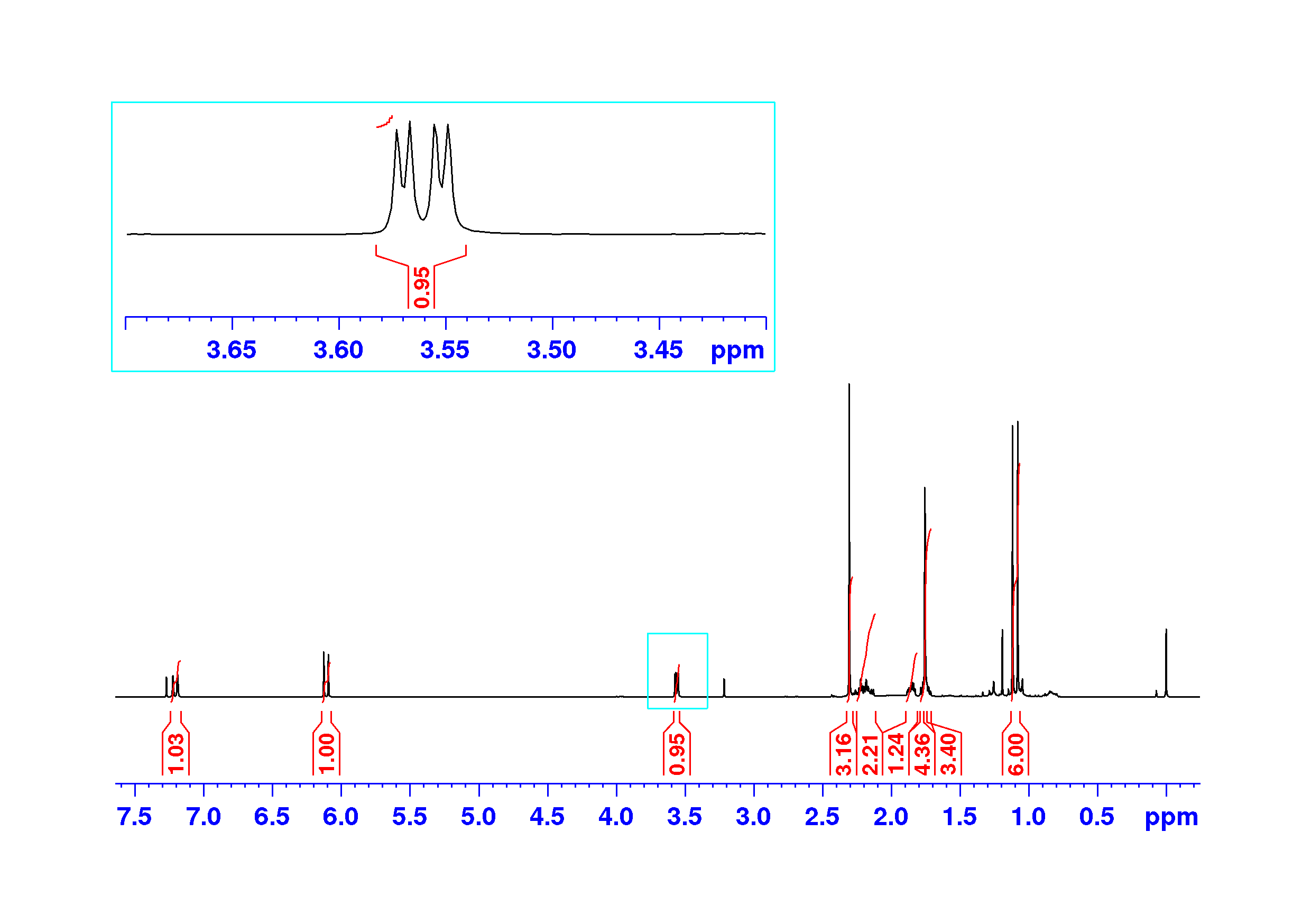


CDCl_3_

TMS


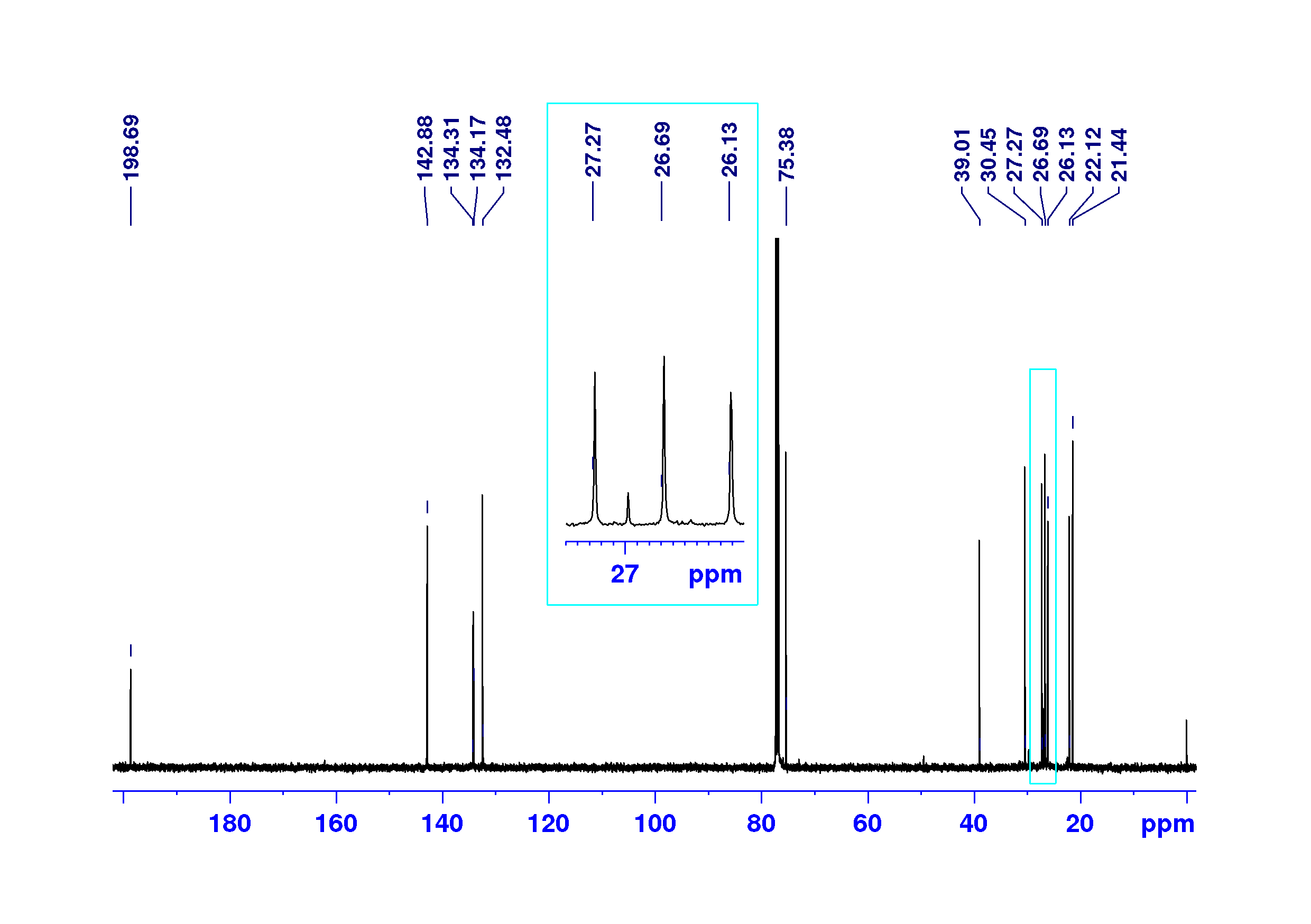


CDCl_3_

TMS

Fig. S11. ^1^H and ^13^C-NMR spectra of 2-hydroxy-β-ionone.

*

IS

β-Caryophyllene

Fig. S12. GC-chromatogram of β-caryophyllene *in vitro* biotransformation. Magenta: negative control; Black: CYP109Q5 wildtype. IS: Internal standard; * Unidentified products.

IS

*

β-damascone

Fig. S13. GC-chromatogram of β-damascone *in vitro* biotransformation. Magenta: negative control; Black: CYP109Q5 wildtype. IS: Internal standard; * Unidentified products.

*

C12

Fig. S14. GC-chromatogram of lauric acid *in vitro* biotransformation. Magenta: negative control; Black: CYP109Q5 wildtype. * Unidentified products.

Diclofenac

4-OH-diclofenac

Fig. S15. HPLC-chromatogram of diclofenac *in vitro* biotransformation. blue: negative control; red: CYP109Q5 wildtype.

*

Testosterone

Fig. S16. LCMS-chromatogram of testosterone *in vitro* biotransformation. red: negative control; blue: CYP109Q5 wildtype. * Unidentified products.

**References**

Bell, S.G., Dale, A., Rees, N.H., and Wong, L.-L. (2010) A cytochrome P450 class I electron transfer system from *Novosphingobium aromaticivorans*. *Appl. Microbiol. Biotechnol.* **86**: 163–175.

Bell, S.G. and Wong, L.-L. (2007) P450 enzymes from the bacterium *Novosphingobium aromaticivorans*. *Biochem. Biophys. Res. Commun.* **360**: 666–672.

Celik, A., Flitsch, S.L., and Turner, N.J. (2005) Efficient terpene hydroxylation catalysts based upon P450 enzymes derived from actinomycetes. *Org. Biomol. Chem.* **3**: 2930–2934.

Chakrabarti, R. and Schutt, C.E. (2001) The enhancement of PCR amplification by low molecular-weight sulfones. *Gene* **274**: 293–298.

Dietrich, M., Eiben, S., Asta, C., Do, T.A., Pleiss, J., and Urlacher, V.B. (2008) Cloning, expression and characterisation of CYP102A7, a self-sufficient P450 monooxygenase from *Bacillus licheniformis*. *Appl. Microbiol. Biotechnol.* **79**: 931–940.

Girhard, M., Klaus, T., Khatri, Y., Bernhardt, R., and Urlacher, V.B. (2010) Characterization of the versatile monooxygenase CYP109B1 from *Bacillus subtilis*. *Appl. Microbiol. Biotechnol.* **87**: 595–607.

Grivel, F. and Larroche, C. (2001) Phase transfer and biocatalyst behaviour during biotransformation of beta-ionone in a two-phase liquid system by immobilised *Aspergillus niger*. *Biochem. Eng. J.* **7**: 27–34.

Hall, E.A. and Bell, S.G. (2015) The efficient and selective biocatalytic oxidation of norisoprenoid and aromatic substrates by CYP101B1 from *Novosphingobium aromaticivorans* DSM12444. *RSC Adv.* **5**: 5762–5773.

Jóźwik, I.K., Kiss, F.M., Gricman, Ł., Abdulmughni, A., Brill, E., Zapp, J., et al. (2016) Structural basis of steroid binding and oxidation by the cytochrome P450 CYP109E1 from *Bacillus megaterium*. *FEBS J.* **283**: 4128–4148.

Khatri, Y., Bernhardt, R., Girhard, M., Hannemann, F., and Urlacher, V.B. (2011) Biocatalyst for hydroxylation and / or epoxidation.

Khatri, Y., Girhard, M., Romankiewicz, A., Ringle, M., Hannemann, F., Urlacher, V.B., et al. (2010) Regioselective hydroxylation of norisoprenoids by CYP109D1 from *Sorangium cellulosum* so ce56. *Appl. Microbiol. Biotechnol.* **88**: 485–495.

Larroche, C., Creuly, C., and Gros, J.-B. (1995) Fed-batch biotransformation of beta-ionone by *Aspergillus niger*. *Appl. Microbiol. Biotechnol.* **43**: 222–227.

Litzenburger, M. and Bernhardt, R. (2016) Selective oxidation of carotenoid-derived aroma compounds by CYP260B1 and CYP267B1 from *Sorangium cellulosum* So ce56. *Appl. Microbiol. Biotechnol.* **100**: 4447–4457.

Lutz-Wahl, S., Fischer, P., Schmidt-Dannert, C., Wohlleben, W., Hauer, B., and Schmid, R.D. (1998) Stereo- and regioselective hydroxylation of alpha-ionone by *Streptomyces* strains. *Appl. Environ. Microbiol.* **64**: 3878–3881.

Ly, T.T.B., Khatri, Y., Zapp, J., Hutter, M.C., and Bernhardt, R. (2012) CYP264B1 from *Sorangium cellulosum* So ce56: a fascinating norisoprenoid and sesquiterpene hydroxylase. *Appl. Microbiol. Biotechnol.* **95**: 123–133.

Ma, M., Bell, S.G., Yang, W., Hao, Y., Rees, N.H., Bartlam, M., et al. (2011) Structural Analysis of CYP101C1 from *Novosphingobium aromaticivorans* DSM12444. *Chembiochem* **12**: 88–99.

Mikami, Y., Watanabe, E., Fukunaga, Y., and Kisaki, T. (1978) Formation of 2S-Hydroxy-beta-ionone and 4-Hydroxy-beta-ionone by microbial hydroxylation of beta-ionone. *Agric. Biol. Chem.* **42**: 1075–1077.

Putkaradze, N., Litzenburger, M., Abdulmughni, A., Milhim, M., Brill, E., Hannemann, F., and Bernhardt, R. (2017) CYP109E1 is a novel versatile statin and terpene oxidase from *Bacillus megaterium*. *Appl. Microbiol. Biotechnol.* **49**: 1–15.

Sode, K., Karube, I., Araki, R., and Mikami, Y. (1989) Microbial conversion of beta-ionone by immobilized *Aspergillus niger* in the presence of an organic solvent. *Biotechnol Bioeng* **33**: 1191–1195.

Urlacher, V.B., Makhsumkhanov, A., and Schmid, R.D. (2006) Biotransformation of β-ionone by engineered cytochrome P450 BM-3. *Appl. Microbiol. Biotechnol.* **70**: 53–59.

Zehentgruber, D., Urlacher, V.B., and Lütz, S. (2012) Studies on the enantioselective oxidation of β-ionone with a whole *E. coli* system expressing cytochrome P450 monooxygenase BM3. *J. Mol. Catal. B Enzym.* **84**: 62–64.

Zhang, A., Zhang, T., Hall, E.A., Hutchinson, S., Cryle, M.J., Wong, L.-L., et al. (2015) The crystal structure of the versatile cytochrome P450 enzyme CYP109B1 from *Bacillus subtilis.* *Mol. Biosyst.* **11**: 869–881.
